# Elastic spheres can walk on water

**DOI:** 10.1038/ncomms10551

**Published:** 2016-02-04

**Authors:** Jesse Belden, Randy C. Hurd, Michael A. Jandron, Allan F. Bower, Tadd T. Truscott

**Affiliations:** 1 Naval Undersea Warfare Center Division Newport, 1176 Howell Street, Newport, Rhode Island 02841, USA; 2 Department of Mechanical and Aerospace Engineering, Utah State University, ENGR 419J, Logan, Utah 84322, USA; 3 School of Engineering, Brown University, 184 Hope Street, Providence, Rhode Island 02912, USA

## Abstract

Incited by public fascination and engineering application, water-skipping of rigid stones and spheres has received considerable study. While these objects can be coaxed to ricochet, elastic spheres demonstrate superior water-skipping ability, but little is known about the effect of large material compliance on water impact physics. Here we show that upon water impact, very compliant spheres naturally assume a disk-like geometry and dynamic orientation that are favourable for water-skipping. Experiments and numerical modelling reveal that the initial spherical shape evolves as elastic waves propagate through the material. We find that the skipping dynamics are governed by the wave propagation speed and by the ratio of material shear modulus to hydrodynamic pressure. With these insights, we explain why softer spheres skip more easily than stiffer ones. Our results advance understanding of fluid-elastic body interaction during water impact, which could benefit inflatable craft modelling and, more playfully, design of elastic aquatic toys.

## Abstract (web summary)

Stone skipping on water is a very classical phenomenon, but the relevant fluid dynamics is more complicated than what is normally expected. Here, Belden *et al*. study the effect of elasticity on the skipping dynamics and show why soft spheres can skip much easier than solid spheres.

## Graphical abstract

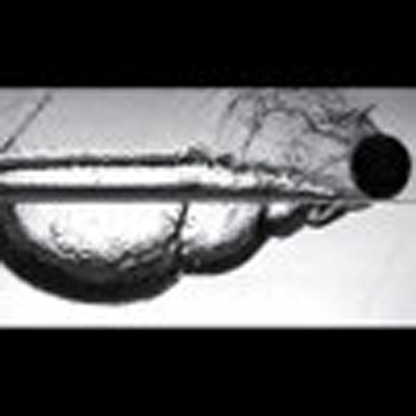

Water-skipping has been studied for centuries with diverse motivations including: the ancient art of stone skipping^1–4^; naval application^5–10^; water-surface craft^11^,^12^; and biological^13^,^14^ and biomimetic^15^ water-walking. While water ricochet of rigid objects has been well studied, the physics underlying the water impact of highly deformable elastic solids remains poorly understood^12^,^16^,^17^. Compliant bodies such as inflatable boats^12^ and elastic aquatic toys^18^,^19^ exhibit behaviour that is not readily explained within the traditional framework for rigid objects. For such elastic bodies, an understanding of the coupling between the material response and hydrodynamic loading is essential in unravelling the overall dynamics.

An object obliquely impacting a water surface with sufficient inertia will carve a cavity on the air–water interface^20^ and experience a pressure-driven hydrodynamic force dependent on object velocity, geometry and orientation^4^,^8^,^21^,^22^,^23^. Water-skipping occurs when the upward vertical component of this force is large enough to lift the object off the water surface^8^. Studies born from naval applications ranging from cannonball skipping tactics^5^,^8^ to the dam-busting Wallis bomb^10^ have revealed an upper bound on the impact angle *β*_o_ (angle between the free-surface and object velocity vector) below which rigid spheres will skip on water^7^,^8^,^10^,^21^. Disk-shaped stones are more amenable to skipping, particularly if one orients the stone at just the right angle^2^. Further research has revealed more details regarding the oblique water impact of these and other canonical rigid body geometries^23^,^24^,^25^,^26^. The referenced ricochet events are dominated by inertia, with negligible contributions from viscous and surface tension forces^4^,^20^. In this regime, the physics of water-skipping also generalize to the water-walking ability of basilisk lizards^13^,^14^ and some birds^14^,^27^, and to surface craft slamming^11^.

In this work, we investigate the skipping of deformable elastic solid spheres on water. We observe that elastic spheres can skip for impact angles nearly three times larger than predicted for rigid spheres. Experiments and numerical modelling show that the spheres deform throughout impact in response to elastic waves propagating in the material. In some cases these elastic waves actually interact with the air–water interface to create nested cavities. We determine how the deformed geometry scales with material properties and initial impact kinematics. Using an analytical model to relate deformation to the hydrodynamic lift force, we identify the mechanisms by which elastic spheres skip so readily on water. Furthermore, we compute the normal and tangential restitution coefficients and find, surprisingly, that they display analogous behaviour to liquid droplets bouncing on inclined liquid films^28^. Based on our findings about single impact events, we explain how elastic spheres are able to achieve multiple successive skips on water.

## Results

### Elastic sphere skipping phenomena

**Figure 1:**
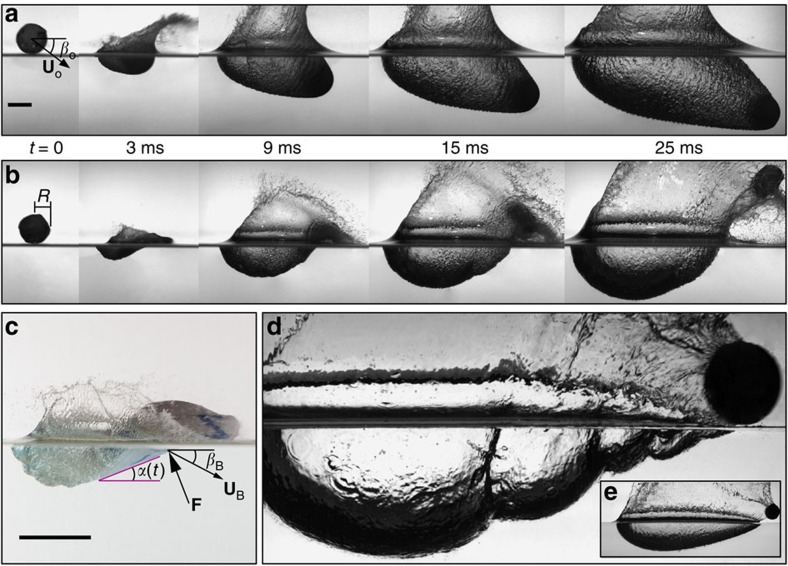
Elasticity alters sphere skipping dynamics. (**a**) High-speed images of an oblique impact show a rigid sphere carving an air cavity into the water as it dives below the surface (*U*_o_=24.3 m s^−1^; *β*_o_=29.6°; *G*=5.66 × 10^5^ kPa; *R*=25.8 mm; and ρ*=*ρ*_s_/*ρ*_w_=0.959). Scale bar, 40 mm. (**b**) A highly compliant elastic sphere significantly deforms upon impact and skips off the surface (*U*_o_=22.0 m s^−1^; *β*_o_=32.0°; *G*=12.3 kPa; *R*=26.2 mm; and ρ*=0.937). (**c**) The deformed sphere resembles a disk-shaped stone oriented at a dynamic attack angle *α*. An inertia-dominated hydrodynamic force **F** acts on the flattened face, which moves with velocity described by **U**_B_ and *β*_B_. Scale bar, 40 mm. (**d**) A surprising consequence of the interaction of sphere vibrations with the liquid interface is the formation of nested air cavities (that is, a matryoshka cavity). (**e**) A rigid sphere can skip if the impact angle does not exceed 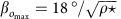, leaving a smooth, asymmetric cavity on the surface (*β*_o_=17.3° for sphere shown).

Prior research has reported an upper bound on the impact angle of 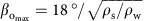 below which rigid spheres (density *ρ*_s_) will skip on water (density *ρ*_w_)^7^,^8^,^10^,^21^. We have found that elastic spheres skip at much larger values of *β*_o_, raising the question of how the elastic response enables this enhanced skipping behaviour. To investigate the mechanisms underlying elastic sphere skipping, we film the water impact of custom-made elastomeric spheres with a high-speed camera viewing from the side. Rigid and elastic spheres having nearly the same radius *R* and density *ρ*_s_ are shown experimentally impacting the water in Fig. 1a,b. Each sphere strikes with approximately the same speed *U*_o_ and impact angle *β*_o_, but the elastic sphere has a shear modulus *G* that is four orders of magnitude smaller than that of the rigid sphere material. Within a few milliseconds after impact, the elastic sphere deforms dramatically and rides along the front of a cavity on the air–water interface before lifting off the surface. By the time the elastic sphere is two diameters above the surface (*t*≈25 ms), the rigid sphere has plunged nearly the same distance below it. The elastic sphere evidently experiences a larger upward vertical force from the water.

The extreme sphere deformation is more evident in Fig. 1c, which shows that the water-contacting surface assumes the shape of a disk with a larger radius than that of the undeformed sphere. The disk is oriented at an attack angle *α* that, unlike for skipping stones^4^, changes in time throughout the impact. Large amplitude oscillations excited by the impact can persist in the sphere after lifting off the surface (Fig. 1b and Supplementary Movie 1) or even while the sphere is still in contact with the water (Fig. 1d and Supplementary Movie 2). In the latter case, the sphere vibrations form a group of nested cavities, or a so-called matryoshka cavity^29^, named after Russian nesting dolls (Fig. 1d). This phenomenon is in contrast to rigid sphere skipping, for which the cavity is asymmetric, but smooth (Fig. 1e).

### Sphere deformation modes

**Figure 2:**
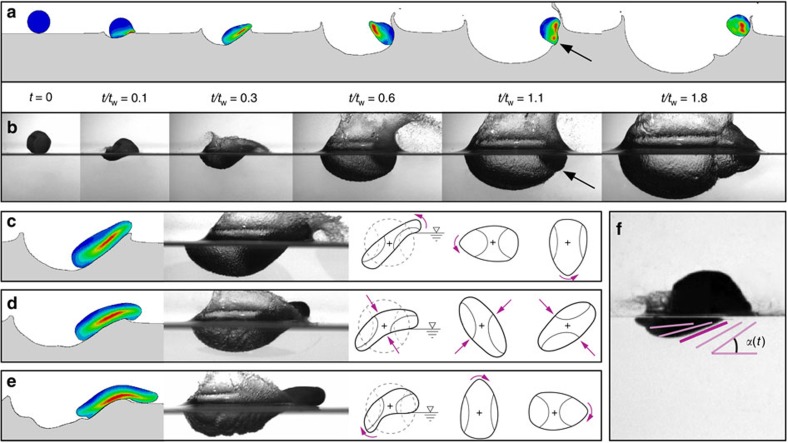
Fluid-structure coupling through sphere deformation modes. (**a**) An Abaqus numerical model captures the skipping behaviour and reveals the local relative strain in the sphere (colour contours). (**b**) Under the same conditions as simulated in (**a**) an experiment shows the formation of a nested cavity (or so-called matryoshka cavity), as also shown by the model. (**a**,**c**) The model reveals an elastic wave that propagates in a counter-clockwise direction around the sphere (classified as deformation mode 1^−^). At time *t*_w_, the elastic wave strikes the air–water interface. Two other deformation modes are observed; (**d**) mode 2: the sphere assumes an ellipsoidal shape with oscillating major and minor axes (Supplementary Movie 1); (**e**) mode 1^+^: an elastic wave propagates in the clockwise direction. (**f**) The attack angle *α* evolves in time in response to the elastic wave propagation (deformation mode 1^−^ is pictured). The purple lines are experimental measurements of *α* taken at 1.33 ms intervals.

To examine the sphere deformations more thoroughly, we implement a fully coupled numerical finite-element model in Abaqus (ref. 30) (see Methods section). Figure 2a shows the results of a numerical simulation carried out with the same sphere material properties (*R*, *G* and *ρ*_s_) and impact conditions (*U*_o_ and *β*_o_) as the experimental test shown in Fig. 2b. The numerics reveal an elastic wave propagating around the circumference of the sphere in a counter-clockwise direction. We classify this type of wave propagation, depicted in the line drawing of Fig. 2c, as vibration mode 1^−^. In some cases this elastic wave impacts the air–water interface at time *t*_w_, thus initiating a matryoshka cavity (as seen numerically and experimentally in Fig. 2a,b). While tempting to attribute these kinematics to rigid body rotation, we find that the elastic wave propagation time *t*_w_ is typically much smaller than the measured period of rigid rotation (see Methods and Supplementary Movie 2).

The Abaqus numerical model predicts two additional vibration modes, generally occurring with increasing impact speed and/or decreasing shear modulus. In mode 2 (Fig. 2d, Supplementary Movie 1), the sphere assumes an ellipsoidal shape with oscillating major and minor axes. In mode 1^+^ (Fig. 2e) an elastic wave again propagates around the circumference of the sphere, but in the clockwise direction. Finally, we observe that the attack angle *α* of the deformed water-contacting face evolves as a result of the elastic wave propagation (Fig. 2a,f and Supplementary Movie 2).

### Skip-enhancing mechanisms

Based on our experimental observations and numerical simulations (Figs 1 and 2), we hypothesize that the elastic response of the sphere enhances skipping through two mechanisms: (1) by taking on the shape of a flat disk with an increased wetted area; and (2) by acquiring a favourable attack angle.

**Figure 3:**
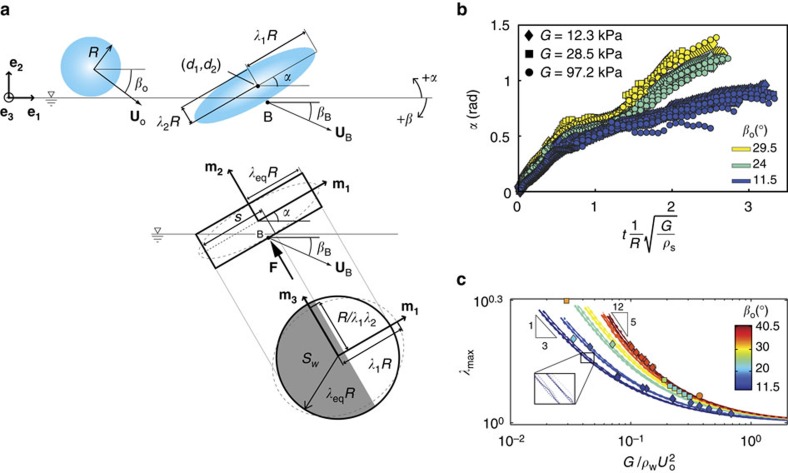
Description of sphere deformation during impact. (**a**) Our analytical model assumes the deformed sphere takes an ellipsoidal shape with principal stretches *λ*_1_, *λ*_2_ and *λ*_3_=1/*λ*_1_*λ*_2_ aligned with the body-fixed {**m**_**1**_, **m**_**2**_, **m**_**3**_} coordinates, respectively. The {**m**_**1**_, **m**_**2**_, **m**_**3**_} coordinate system is inclined at attack angle *α* relative to the free-surface and has its origin at the sphere's centre of mass (*d*_1_,*d*_2_). To model the hydrodynamic force **F**, we represent the sphere as a circular disk with radius *λ*_eq_*R* and assume that **F** acts at the point *B* on the water-contacting face, which moves with velocity described by **U**_B_ and *β*_B_. (**b**) The attack angle *α* is measured from simulations using our Abaqus numerical model for spheres undergoing mode 1^−^ deformations, with properties *R*=25.4 mm, ρ*=1.05 and varying shear modulus; sample *α* measurements are shown in Fig. 4b–d. For each *β*_o_, the attack angle is collapsed by the dimensionless time 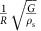, which is proportional to the speed of mode 1^−^ elastic waves propagating through the characteristic distance *R*. These attack angle data are fed into our analytical model (see Methods section). (**c**) The simulations also show the dependence of *λ*_max_ on 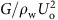, where *λ*_max_ is the maximum value of *λ*_eq_ achieved during impact (individual marker shapes same as for (**b**)). The numerical results agree with predictions from our analytical model (the zoomed region shows the nine different line styles corresponding to analytical results for nine different spheres: dashed lines *R*=20.1 mm; solid lines *R*=26.2 mm; dash-dot lines *R*=48.8 mm; line width indicates *G*: thin *G*=12.3 kPa; middle *G*=28.5 kPa; and thick *G*=97.2 kPa). In the limit of small *G*, we find 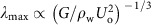 (valid for shallow *β*_o_). For steeper *β*_o_, we find 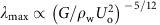 for decreasing 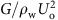.

To connect the suggested skip-enhancing mechanisms to the vertical force acting on the sphere, we propose an analytical model of the coupled fluid-structure interaction. The sphere is idealized as an incompressible, neo-Hookean hyperelastic solid^31^ with shear modulus *G*, radius *R* and density *ρ*_s_. During water impact, the sphere deforms into a disk-shaped ellipsoid inclined at attack angle *α*(*t*) to the water surface (Fig. 3a). A set of equations can then be written for the motion of the centre of mass (COM) and sphere deformation in terms of general forces and tractions acting on the body (see Methods section).

To couple the sphere response to the fluid loading, we extend an existing hydrodynamic force model for circular disk-shaped skipping stones^4^ by approximating the water-contacting face as a circular disk with radius *λ*_eq_*R*, where *λ*_eq_ approximates the sphere deformation (see Fig. 3a and Methods section). The force is modelled as


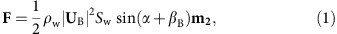


where **U**_B_ and *β*_B_ describe the velocity of the water-contacting face and the wetted area *S*_w_ is proportional to (*λ*_eq_*R*)^2^. The equation for the vertical motion of the COM is then


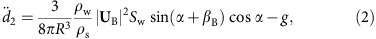


where *d*_2_ is the vertical coordinate of the COM and *g* is gravity. We can now predict how the hypothesized mechanisms relate to skipping. First, a larger area *S*_w_ increases the magnitude of the hydrodynamic force term in equation 2. For a neo-Hookean material, *S*_w_ increases with decreasing shear modulus *G* for a given applied compressive stress^31^. Second, a smaller attack angle increases cos *α* thereby increasing the vertical force component that lifts the sphere off the surface. We note that the governing equations for the sphere deformation predict a steady-state solution in which the attack angle evolves as 
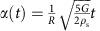
 in response to a circumferential wave propagating in mode 1^−^ (see Supplementary Note 1). While the sphere deformation during impact is not steady-state, we nonetheless expect *α* to be governed by the speed of elastic waves propagating in the sphere through the characteristic distance *R* such that 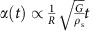. Therefore, we predict that a more compliant sphere (smaller *G*) will assume a smaller rate of change of *α*, thereby increasing the upward vertical force that enables skipping.

Numerical simulations verify the expected dependence of these two mechanisms on *G*. Measurements from the Abaqus results show that the rate of change of the attack angle scales as 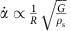 for mode 1^−^ deformations (Fig. 3b). Additionally, we find that the maximum value of *λ*_eq_ achieved during impact, which we call *λ*_max_, increases with a decrease in the dimensionless term 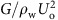 (Fig. 3c), which is the ratio of material stiffness to hydrodynamic pressure. Therefore, a smaller *G* yields a larger stretch and larger wetted area, as well as a smaller rate of change of *α*, as predicted.

**Figure 4:**
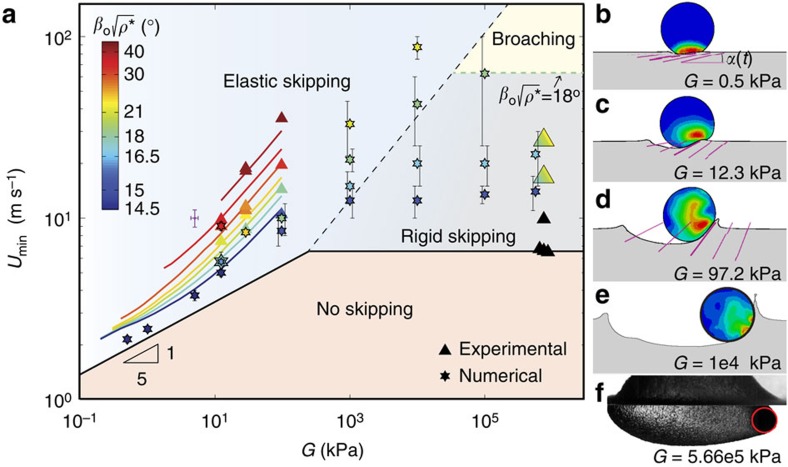
The effect of material stiffness on skipping behaviour. (**a**) Below a threshold stiffness (*G*≈10^3^−10^4^ kPa, depending on *β*_o_), the elastic response of the sphere affects skipping. In this elastic regime, the minimum speed required to skip *U*_min_ decreases monotonically with decreasing shear modulus *G*, as shown by both experimental and numerical results (triangle and star markers, respectively). In the limit of small *G* we expect *U*_min_∝*G*^1/5^, which is confirmed by the numerics. As stiffness increases above *G*≈10 kPa, *U*_min_ deviates from this relation as larger *G* augments the rate of change of *α*, thereby reducing the upward vertical force component. Our analytical model also captures this change in behaviour (solid coloured lines). For shallow impact angles, *U*_min_ becomes insensitive to shear modulus for *G*>≈10^3^ kPa, indicating that rigid sphere skipping behaviour is recovered. The transition between the elastic and rigid skipping regime occurs at larger *G* as *β*_o_ increases. In the rigid skipping regime, *U*_min_ is very sensitive to *β*_o_ (also evident in Supplementary Fig. 1). The lower bound of the rigid skipping regime is inferred from the dark triangle symbols, which occur for experiments with 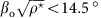. The coloured triangle markers at *G*=5.66 × 10^5^ kPa result from experiments where the colour gradient on the marker indicates the uncertainty in 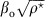. The upper bound on the rigid regime corresponds to 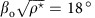; prior literature suggests that for 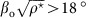 spheres may broach (that is, completely immerse before exiting), but not skip8. (**b**–**d**) Numerical simulations show that increasing *G* results in a larger rate of change of *α* (purple lines) in the elastic skipping regime. Each image shown is 6 ms after impact and the interval between *α* measurements is 2 ms. (**e**) As shear modulus increases into the rigid regime, the sphere deformation is negligible (black outline is undeformed sphere contour). (**f**) We have observed broaching for rigid spheres impacting with 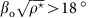. Sphere properties for data in (**a**): rigid sphere experiments, *R*=25.8 mm, ρ*=0.959, *G*=5.66 × 10^5^ kPa; all other data markers, *R*=26.2 mm, ρ*=0.937 for *G*≤12.3 kPa and ρ*=1.03 for *G*>12.3 kPa. The purple error bars are characteristic for experimental data. The numerical error bars represent ±1/2 of the difference in *U*_o_ between the skipping and non-skipping cases used to compute *U*_min_; error bars are offset for clarity.

To confirm that these mechanisms indeed enhance skipping, we perform experiments and simulations over a range of impact conditions and sphere properties and measure the minimum impact speed required to skip *U*_min_, as a function of *G* (Fig. 4). Above a certain value of *G* (≈10^3^−10^4^ kPa, depending on *β*_o_), we recover the rigid skipping regime, in which *U*_min_ is independent of shear modulus but is very sensitive to *β*_o_. For rigid spheres impacting above 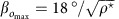, where ρ*=*ρ*_s_/*ρ*_w_, prior research suggests spheres may broach (that is, become completely submerged before exiting), but not skip^8^ (Fig. 4f). For stiffness values below the rigid regime, the elasticity of the sphere becomes important and *U*_min_ decreases monotonically with decreasing shear modulus. Our analytical model accurately predicts the experimental and numerical results in this regime. The minimum speed is also much less sensitive to *β*_o_ in the elastic skipping regime and as a result we observe skipping at impact angles nearly three times larger than predicted for rigid spheres (Fig. 4 and Supplementary Fig. 1).

While our results show that reducing shear modulus has the predicted effect on wetted area and attack angle (Fig. 3b,c), it is unclear whether one or both of these mechanisms are responsible for the observed improved skipping performance. To isolate the mechanisms we consider the limiting case of small *G*, for which 
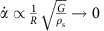
 and thus cos *α*≈1 over typical impact timescales. The expected dependence of *U*_min_ on *G* in this limit can be rationalized by scaling analysis of equation 2 (see Methods and Supplementary Note 2), which gives


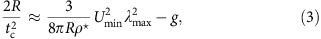


where *t*_c_ is the collision time (that is, time in which the sphere is in contact with the water). For threshold skipping cases, we expect the characteristic acceleration 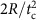 to be small compared with gravity and thus, to first order, 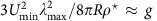. Furthermore, in the small *G* limit our numerical modelling shows that 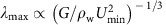 (Fig. 3c). Applying this dependence and solving for *U*_min_, we find 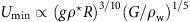. Figure 4a shows that *U*_min_ approaches the *G*^1/5^ relation in the limit of small *G*, indicating that the only mechanism by which reducing shear modulus enhances skipping in this limit is through the increased wetted area. However, for larger *G* (>≈10 kPa), *U*_min_ deviates from the *G*^1/5^ relation as the coupling between shear modulus and *α* becomes important (Fig. 4a–d). As stiffness continues to increase, ultimately the amplitude of the deformations (affecting both *S*_w_ and *α*) become negligible and the sphere is effectively rigid (Fig. 4a,e). Consequently, we conclude that decreasing shear modulus below this rigid boundary causes an increase in the upward vertical force that promotes skipping through both of the hypothesized mechanisms, save for the limit of small *G* where decreasing shear modulus only affects lift by increasing *S*_w_.

### Skipping regimes

**Figure 5:**
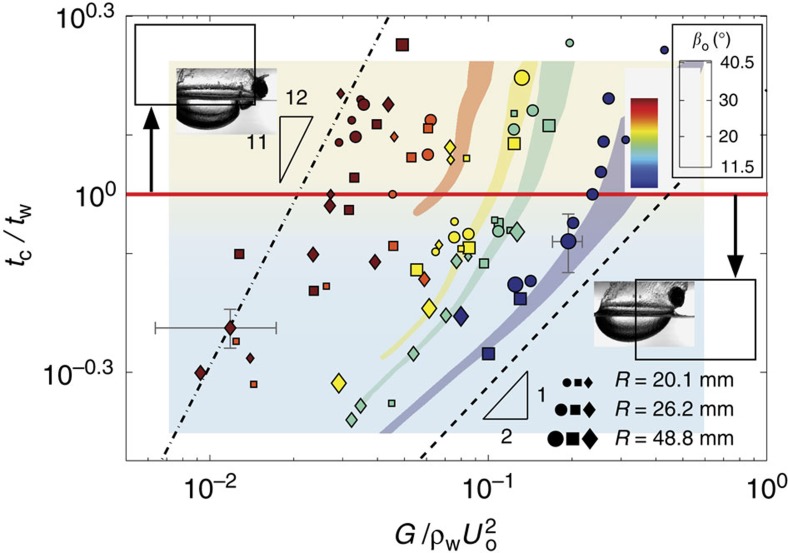
Matryoshka cavities depend on two timescales. When conditions produce impacts in which the collision time *t*_c_ is longer than the elastic wave propagation time *t*_w_ (that is, *t*_c_/*t*_w_>1) matryoshka cavities form. Experiments show that the timescale ratio depends on 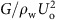 and *β*_o_, with seemingly minimal dependence on *R* (marker size indicates *R*; marker shapes indicate same *G* as for Fig. 3b). The four coloured patches result from calculations using our analytical model with the same material properties as for the experiments. Varying *R* in the model over the range experimentally tested results in the spread in the patches, which is small for *t*_c_/*t*_w_<1. For shallow impact angles, scaling analysis predicts 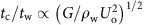 (dashed line), while for steep impact angles we expect 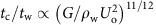 (dash-dot line). These limiting trends capture the general evolution shown by the experimental data. Characteristic error bars are shown.

As the impact events devolve from clear skipping to water entry, we observe a transitional regime characterized by a matryoshka cavity, in which the sphere still skips (Fig. 1d; Supplementary Movie 2). The matryoshka phenomenon occurs when the total contact time of the sphere with water *t*_c_ is longer than the wave time *t*_w_ associated with mode 1^−^ elastic wave propagation, such that *t*_c_/*t*_w_>1. We define *t*_w_ as the time from impact until the circumferential elastic wave strikes the air–water interface (Fig. 2a,b). Experiments over a range of sphere properties and impact conditions reveal that *t*_c_/*t*_w_ is governed by the impact angle *β*_o_ and the ratio 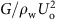 (Fig. 5). The dependence on these terms can be rationalized by scaling analysis with *U*_o_ replacing *U*_min_ in equation 3. When *t*_c_≈*t*_w_, the characteristic sphere acceleration is much greater than *g* such that 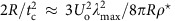 and we find 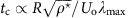 (see Methods section). Furthermore, based on the propagation speed of mode 1^−^ elastic waves, we expect 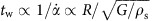, which is confirmed experimentally (Supplementary Fig. 2). Combining the scaling for each time gives 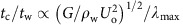. We can now examine the evolution of the timescale ratio in the vicinity of the transitional regime in the limit of shallow (*β*_o_→0) and steep impact angles, making use of the relationship between *λ*_max_ and 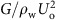 shown in Fig. 3c. Here, we consider steep impact angles to be 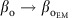, where 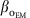 is the maximum impact angle at which we have observed elastic sphere skipping 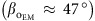. In the shallow *β*_o_ limit, *t*_c_/*t*_w_≈1 occurs at values of 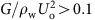, for which *λ*_max_→1 (Fig. 3c); thus, we anticipate 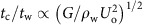 for shallow angles. For steep *β*_o_, the transitional regime occurs for 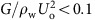, for which 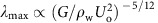 (Fig. 3c), and we expect 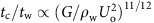. These limiting relations capture the evolution of *t*_c_/*t*_w_ observed experimentally (Fig. 5) and provide insight into the differences observed at different impact angles. We see that for steeper *β*_o_, the sphere deformation has a larger effect on the collision time, which gets manifested as a higher sensitivity of *t*_c_/*t*_w_ to 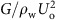.

**Figure 6:**
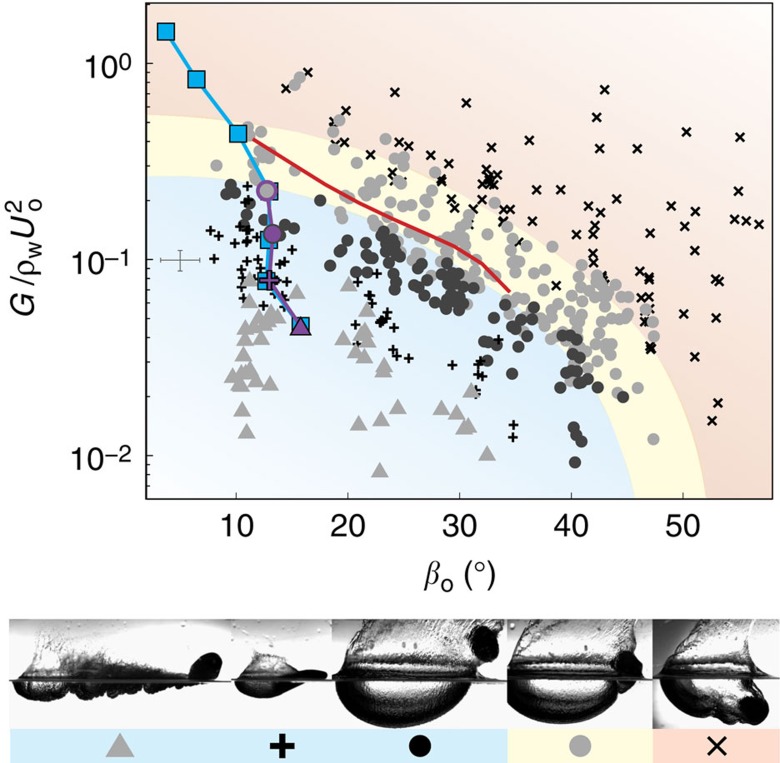
Classification of impact phenomena for single and multiple skip events. Experiments show that all vibration modes and accompanying skipping behaviour are classified by the ratio of material stiffness to hydrodynamic pressure 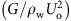 and *β*_o_ (triangles: mode 1^+^; crosses: mode 2; black circles: mode 1^−^ skipping; grey circles: mode 1^−^ transitional; x-marks: water entry). The experimental data shown are for all three elastic sphere radii. The coloured regions provide a visual boundary of the skipping (blue), transitional (yellow) and entry (red) regimes. Our analytical model accurately predicts the boundary between mode 1^−^ skipping and transitional events; the red line is found from the intersection of the analytical results with *t*_c_/*t*_w_=1 in Fig. 5. Given initial conditions of the first skip, our analytical model also predicts successive impact types in a multiple skip trajectory (blue squares). A multiple skip experiment validates these predictions within the limits of our test facility (purple markers; *R*=26.2 mm, *G*=12.3 kPa, ρ*=0.937). Images from the multiple skip experiment are shown in Fig. 8. Characteristic error bars are shown in grey.

Based on our findings regarding the transitional skipping regime, we hypothesize that the same dimensionless parameters (that is, *β*_o_ and 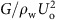) govern all deformation modes and associated skipping behaviour. An empirical regime diagram indeed shows that these parameters classify all observed skip types (Fig. 6). Mode 1^+^ is promoted by shallow *β*_o_, large *U*_o_ and/or small *G*. As stiffness becomes large relative to hydrodynamic pressure, the vibration type traverses the mode 2 and mode 1^−^ regimes. Our analytical model correctly predicts the boundary between the mode 1^−^ skipping (*t*_c_/*t*_w_<1) and mode 1^−^ transitional (*t*_c_/*t*_w_>1) regimes (marked by red line on Fig. 6). A small, radius-dependent overlap region exists between the transitional regime and water entry. Finally, we have observed that the vibration mode associated with the transitional and water entry regimes is exclusively mode 1^−^.

### Skipping sphere rebound

**Figure 7:**
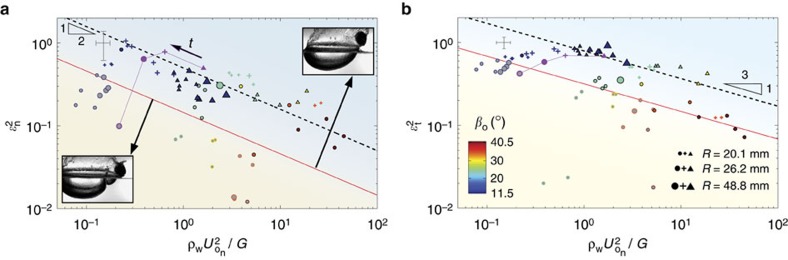
Restitution coefficients of elastic spheres skipping on water. Experiments reveal the dependence of the square of the normal (**a**) and tangential (**b**) restitution coefficients on 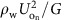 (grey-filled symbols denote mode 1^−^ transitional skips; marker shapes same as for Fig. 6; ρ*=1±0.08). We find that 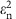 and 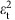 scale with 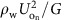 in the same way that for bouncing liquid drops^28^ they scale with 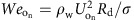. Thus, shear modulus *G* for skipping elastic solid spheres plays the role of the Laplace pressure *σ*/*R*_d_ for bouncing liquid droplets. Because rebound is more efficient in the tangential direction, *β*_o_ decreases (thus becoming more favourable) with each skip. In addition, both restitution coefficients increase with decreasing impact speed until the sphere enters the mode 1^−^ skipping and transitional regimes. These two effects combine to enable multiple skip trajectories (Figs 6 and 8, Supplementary Movie 3). The demise of a multiple skip event starts when *t*_c_/*t*_w_>1 and a matryoshka cavity forms (below red lines); 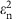 and 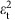 decrease rapidly when this occurs. This interpretation is confirmed by a multiple skip experiment; the purple markers on each plot correspond to the multiple skip experimental data shown in Figs 6 and 8.

We quantify the rebound characteristics of skipping spheres by computing the normal and tangential coefficients of restitution, 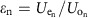 and 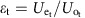, where **U**_*e*_ is the exit velocity and *n* and *t* refer to components normal and tangential to the free-surface, respectively. Here, we find some interesting similarities between skipping elastic spheres and liquid droplets bouncing on inclined liquid layers^28^. Following the work on bouncing liquid droplets, we plot 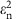 and 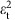 as a function of 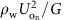 (Fig. 7), which is equivalent to the normal Weber number 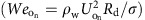 with shear modulus *G* replacing the Laplace pressure *σ*/*R*_d_, where *R*_d_ is the droplet radius and *σ* is surface tension of the liquid droplet. Interestingly, we find 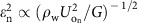 and 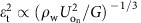, which are the same scaling relations found for liquid droplets bouncing on inclined liquid layers^28^. The restitution coefficients deviate from these trends when *t*_c_/*t*_w_>1. In these cases, sphere vibrations interact with the water via the matryoshka cavity causing a significant decrease in bouncing efficiency. For both elastic skipping spheres and bouncing droplets, the restitution coefficients are always less than one as part of the initial translational kinetic energy goes into post-impact vibrations in the sphere or droplet^28^,^32^.

We build on the bouncing droplet analogy to speculate on the lower bound of validity of the *U*_min_∝*G*^1/5^ scaling relation in the limit *G*→0 (Fig. 4). When the relative magnitude of droplet surface tension becomes small for liquid droplets impacting liquid layers, bouncing does not occur and the droplet completely merges with the liquid layer^28^. We conjecture about a similar limit for elastic spheres with *G*→0 and impacting with 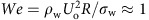, where *σ*_w_ is the surface tension of water. In this limit, we expect the surface tension force from the water to act prominently on the sphere^33^ and to inhibit sphere reformation, thus preventing recovery of translational kinetic energy from deformational potential energy during impact. As a result, we hypothesize that sphere skipping would ultimately cease in this limit. We anticipate these dynamics would become relevant when *G*≈*σ*_w_/*R* (see Supplementary Fig. 3 and Supplementary Note 3). To validate these predictions is beyond the scope of the present work.

## Discussion

**Figure 8:**
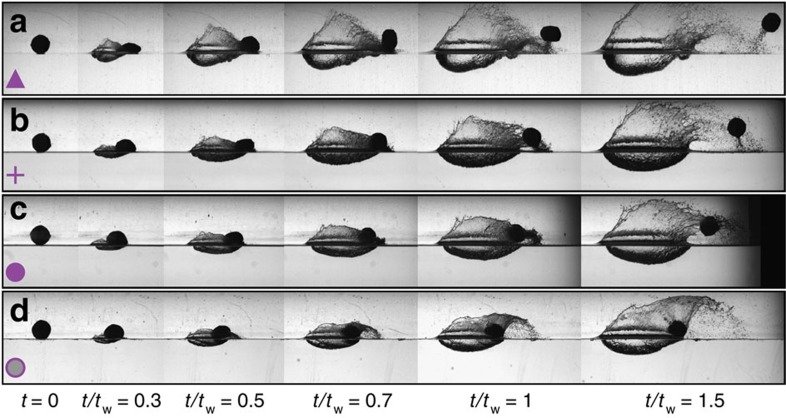
An elastic sphere experiences several modes in a multiple skip event. These image sequences correspond to the four successive experimental impacts shown as purple symbols in Figs 6 and 7. (**a**) The first skip displays mode 1^+^ behaviour. (**b**) The impact angle *β*_*o*_ is reduced for the second skip, which is in the mode 2 regime. (**c**) The third impact (mode 1^−^ skipping) occurs with approximately the same value of *β*_*o*_, but leads to reduced 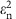 and 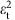. (**d**) The last skip shows mode 1^−^ transitional behaviour, and results in such small values of the restitution coefficients that the ensuing impact ends in water entry (not shown). All times are normalized by the wave time computed from the mode 1^−^ transitional skip (**d**).

Perhaps the most mesmerizing manifestation of elastic sphere water impact is continual skipping across water (Supplementary Movie 3). To confirm that our physical description of a single skip generalizes to multiple skip events, we predict the placement of successive impacts on the regime diagram (Fig. 6). An experimental investigation using isolated water tanks validates the predictions and shows the sphere traversing through the vibration modes with each impact (Fig. 8). As to how repeated skipping is sustained over very long skipping trajectories (Supplementary Movie 3), we gain insight from the behaviour of the restitution coefficients (Fig. 7). First, *ɛ*_t_ is consistently larger than *ɛ*_n_, which causes *β*_o_ to decrease and thus become more favourable with each skip. Second, the restitution coefficients actually become larger as 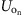 decreases, until the sphere enters the mode 1^−^ skipping and transitional regimes. Therefore, one could say it becomes easier to skip with every skip.

While toy elastic balls may bestow upon the casual sportsman the ability to break the world stone skipping record (88 skips by K. Steiner, Guinness World Records), we believe the physics underlying the elastic sphere impact are common to the large deformation hydroelastic response of surface-riding and skipping compliant bodies. Models of these structures, such as inflatable boats, typically ignore extreme elastic deformation even though it is known to affect drag, stability and slamming loads^12^. The mechanisms of form and force augmentation, as well as the secondary vibration-induced fluid interactions that we have revealed, can be exploited for functional advantage and incorporated into higher-fidelity hydroelastic models.

## Methods

### Sphere fabrication and material properties

In order to control material properties, custom elastomeric spheres were fabricated from a high performance platinum-cure silicone rubber called Dragon Skin produced by Smooth-On, Inc., which consists of two liquid constituent parts. Once the two constituents are mixed, the material sets without requiring heat treatment. The shear modulus was varied by adding a silicone thinner to the mixture before setting, which reduces the material shear modulus by decreasing the polymer cross-linking density. Sphere materials with three different shear moduli were fabricated by adding 0, 1/3 and 1/2 parts thinner by mass ratio. Before setting, the liquid mixture was placed in a vacuum chamber to remove any entrained air. For our experiments, spheres were fabricated by curing the liquid mixture in smooth, machined aluminium moulds to produce spheres with three different radii: 20.1±0.8 mm; 26.2±0.8 mm; and 48.8±0.9 mm. A rigid sphere with *R*=25.8±1 mm was fabricated from Nylon DuraForm PA using selective laser sintering. The uncertainty on each sphere radius represents the 95% confidence interval based on several independent measurements. A thin Lycra casing was loosely fitted around each sphere in order to prevent undesired particles from adhering to the surface and to reduce the friction between the sphere and the launching mechanism from which it was fired.

The silicone rubber was so compliant that traditional uniaxial ‘dogbone' testing on our Instron machine was not feasible as the forces generated were too small to be reliably measured. To overcome this, we performed a test in which the spheres were compressed on the Instron to generate a quasi-static force-displacement curve. This test set-up was then numerically modelled in Abaqus with the sphere material described by a neo-Hookean hyperelastic constitutive model, parameterized by the shear modulus *G*31. We then varied *G* to find the value that produced the best fit between the numerically simulated and experimentally measured force-displacement curves. The elastomeric spheres used in our experiments had shear moduli of 97.2, 28.5 and 12.3 kPa corresponding to 0, 1/3 and 1/2 parts thinner, respectively. According to the manufacturer of the rigid sphere (3D Systems—Quickparts Solutions), the elastic modulus of the selective laser sintering Nylon DuraForm PA material is 1.59 × 10^6^ kPa, which—assuming a Poisson's ratio of 0.4—gives a shear modulus of *G*=5.66 × 10^5^ kPa.

### Sphere skipping experiments and data processing

Spheres were launched at the water surface from a variable-angle, pressure-driven cannon consisting of a pressure chamber for compressed air, a sliding cylindrical piston and interchangeable barrels. Sliding the cylindrical piston allowed air to flow from the pressure chamber into the barrel, thus forcing the sphere to accelerate out of the barrel and strike the water surface with impact speed *U*_o_ and angle *β*_o_. Impact events were illuminated with diffuse white back lighting and filmed with either NAC GX-3 or Photron SA3 high-speed cameras acquiring at 1,000–2,000 frames per second (fps).

The impact speed *U*_o_ and angle *β*_o_ were measured from images of the sphere before water impact using a cross-correlation algorithm. The mean uncertainties on *U*_o_ and *β*_o_ are ±1.09 m s^−1^ and ±1.75°, respectively (computed at 95% confidence level). The same algorithm was used to measure the exit speed and angle of the sphere after lifting off the surface. Also, the rigid body rotation of the sphere was estimated after skipping by tracking reference markers on the exterior of the Lycra casing. It was found for mode 1^−^ skip types that the rotational kinetic energy was typically <8% of the translational kinetic energy after water exit. Furthermore, for these impacts the wave time *t*_w_ was typically <30% of the period of rigid body rotation measured after skipping. The collision time *t*_c_, wave time *t*_w_ and vibration mode classification were all determined from manual inspection of the high-speed images. The minimum skipping speed *U*_min_ was found experimentally by performing successive experiments with identical conditions but with increasing speed until the sphere skipped.

### Abaqus numerical model

Details of the Abaqus numerical model of the elastic sphere impact are contained in reference^30^; clarifications relevant for the present work are summarized here. The finite-element model uses the built in coupled euler–lagrange functionality of Abaqus/Explicit, which couples the contact region between the Lagrangian (sphere) and Eulerian (fluid) domains using a penalty method. Direct numerical simulation of the compressible Navier–Stokes equations is performed in the Eulerian domain. For the solid, conservation of momentum is solved with an incompressible neo-Hookean constitutive model describing the sphere. For all numerical model results presented herein, the mesh consists of eight-noded Eulerian hexahedral elements with spatial resolution of 3 mm. The sphere radius *R*, density *ρ*_s_ and shear modulus *G*, as well as the impact speed *U*_o_ and angle *β*_o_ were set to match experimental values. The three-dimensional computational domain consists of a water tank (length=30 *R*, depth=6.5 *R*) with a symmetry plane coinciding with the plane of motion. For the numerical results presented in Fig. 4a, *U*_min_ is the average of the impact speeds for a skipping case and the non-skipping case with the nearest *U*_o_. The numerical marker error bars reported in Fig. 4a represent ±1/2 of the speed difference between the two cases.

### Analytical model of elastic sphere skipping

An approximate analytical approach to modelling the impact between a compliant elastomeric sphere and a fluid surface is outlined (for a complete derivation, see Supplementary Note 1). Here, we describe the sphere deformation and motion using a set of reduced, scalar generalized coordinates that are governed by a system of ordinary differential equations (ODEs). We begin by defining a fixed Cartesian coordinate system {**e**_**1**_, **e**_**2**_, **e**_**3**_} (Fig. 3a). We assume the sphere moves only in the **e**_**1**_−**e**_**2**_ plane and undergoes no rigid body rotation. The sphere deformation is first described by a rigid displacement **d** that describes the motion of the COM in terms of the generalized coordinates *d*_1_, *d*_2_. This is followed by a volume preserving stretch **V** that deforms the sphere into an ellipsoid. The coordinates of a material particle before deformation (**x**) and after deformation (**y**) are related by **y**=**d**+**Vx**. The velocity and acceleration fields follow as 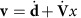 and 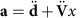. The stretch **V** and its time derivatives 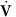, 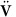 can be written in terms of the principal stretches *λ*_1_, *λ*_2_ and *λ*_3_=1/*λ*_1_*λ*_2_, which are aligned with the body-fixed {**m**_**1**_, **m**_**2**_, **m**_**3**_} coordinates, respectively (see Fig. 3a and Supplementary Note 1). The {**m**_**1**_, **m**_**2**_, **m**_**3**_} coordinate system is inclined at attack angle *α* relative to the free-surface. We introduce a virtual velocity field 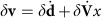 where the kinematic variables are associated with virtual rates of change 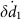, 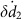, 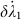, 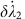, 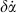 about the current (deformed) state. The governing equations for the generalized coordinates *d*_1_, *d*_2_, *λ*_1_, *λ*_2_ and *α* are obtained from the principle of virtual work (that is, weak form of the momentum conservation equation)^31^


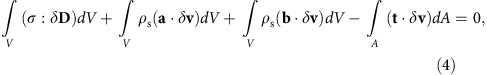


where *σ* is the Cauchy stress tensor, **D** is the stretch rate, **b** are body forces, **t** are traction forces and *V* and *A* denote integration over the volume and surface of the deformed solid, respectively. The term involving the external traction can be re-written as


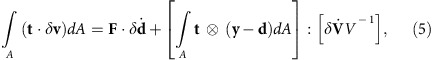


where **F** represents the resultant hydrodynamic force acting on the solid and the second term on the right-hand side represents the virtual power associated with a force dipole tending to distort the elastomer. Expressing equations 4 and 5 in terms of the generalized coordinates (see Supplementary Note 1) and then setting each of 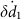, 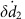, 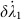, 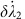, 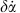 to be non-zero in turn yields a set of coupled second order nonlinear ODEs in terms of general forces and tractions:


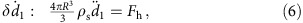



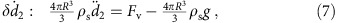



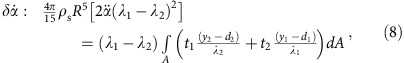



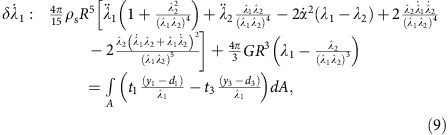



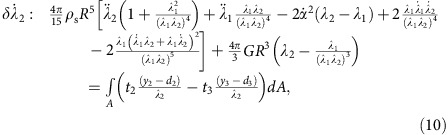


where *F*_h_ and *F*_v_ are the horizontal and vertical force components in {**e**_**1**_, **e**_**2**_, **e**_**3**_} coordinates, respectively, and *t*_*i*_ and *y*_*i*_ are the components of the traction vector and position vector on the surface of the ellipsoid in {**m**_**1**_, **m**_**2**_, **m**_**3**_} coordinates, respectively.

Next, we propose an approximate model for the hydrodynamic forcing on the sphere. It is beyond the scope of this work to derive an analytical expression for the dynamic pressure distribution over the wetted sphere surface. Rather, our goal is to generate a simplified model that captures the first-order sphere motion and deformation during an oblique water impact. Thus, the hydrodynamic force **F** is computed using equation 1, which follows from work on skipping stones^4^ by considering the deformed sphere to be a circular disk. The disk radius is described by an equivalent principal stretch *λ*_eq_ computed by equating the area of the equivalent circular disk with the cross-sectional area of the deformed sphere in the **m**_**1**_-**m**_**3**_ plane, *π*(*λ*_eq_*R*)^2^=*π*(*λ*_1_*R*)(*R*/*λ*_1_*λ*_2_), which gives 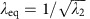. Furthermore, *S*_w_, **U**_B_ and *β*_B_ can be written in terms of the generalized coordinates describing the sphere deformation (see Supplementary Note 1). Without describing the pressure distribution, we cannot specify the centre of pressure and, thus, cannot define the *y*_1_ coordinate at which the traction vector acts in equation 8, which governs the attack angle *α*. To overcome this, we determine *α* from our Abaqus numerical model (Fig. 3b). With *α* prescribed, the remaining ODEs for *d*_1_, *d*_2_, *λ*_1_ and *λ*_2_ (equations 6, 7, 9 and 10) can be solved without further simplification. Inserting the hydrodynamic force yields equation 2 and the remaining ODEs are:


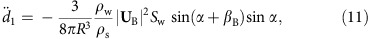



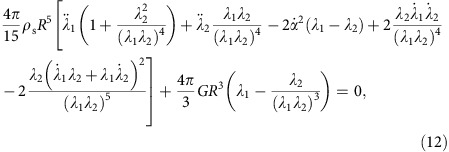



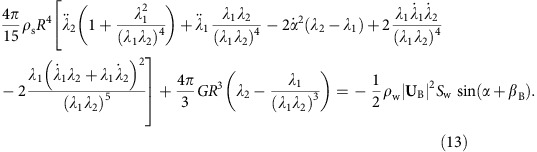


Equations 2 and 11, 12, 13 are solved using a fourth-order Runge–Kutta solver in Matlab, with *α* and 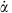 prescribed based on the Abaqus numerical model results.

### Scaling analysis

Using *U*_min_ as the characteristic speed, (*λ*_max_*R*)^2^ as the characteristic wetted area and 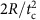 as the characteristic acceleration, equation 2 can be estimated as


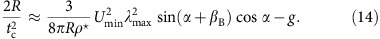


For cases that barely skip, we have observed *t*_c_≈*O*(10^−1^)s, which gives a characteristic acceleration of 
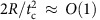
m s^−2^ (with *R*≈*O*(10^−2^)m). Therefore, in the case of the minimum impact speed, we expect gravity to be an order of magnitude larger than the sphere acceleration such that


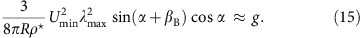


In the limit of small *G*, simulations show that sin (*α*+*β*_B_) cos *α* is of order 1 (see Supplementary Note 2) and 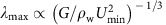 (see Fig. 3c). Thus, equation 15 leads to


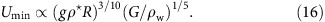


To determine how *t*_c_/*t*_w_ evolves, we consider a scaling analysis of equation 14 when *t*_c_≈*t*_w_, which is typically *O*(10^−2^)s for our experiments. We find the characteristic acceleration 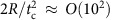
m s^−2^ and thus can neglect gravity. Replacing *U*_min_ with *U*_o_ in equation 14 and solving for *t*_c_ gives 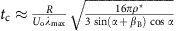. Simulations show that 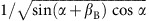 is order 1 (see Supplementary Note 2). Thus, the collision time scales as


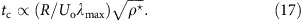


## Additional information

**How to cite this article:** Belden, J. *et al*. Elastic spheres can walk on water. *Nat. Commun.* 7:10551 doi: 10.1038/ncomms10551 (2016).

## Supplementary Material

Supplementary Information Supplementary Figures 1-3, Supplementary Notes 1-3 and Supplementary References

Supplementary Movie 1 Elastic spheres deform dramatically upon water impact. An elastic sphere impacting the water surface assumes a favorably oriented disk-like shape that promotes skipping. Large amplitude elastic oscillations initiated upon impact persist after the sphere lifts off the water surface. This video demonstrates vibration mode 2 in which the sphere assumes an ellipsoidal shape with oscillating major and minor axes.

Supplementary Movie 2 Sphere vibrations induce a matryoshka cavity. In what we refer to as the transitional impact regime, elastic spheres carve nested disturbances on the surface forming a matryoshka cavity. When viewed from the side (left hand video frame), the disturbances appear to result from rigid body rotation of the sphere. However, when viewed in a time-synced top view (right hand video frame), the stitching on the sphere casing indicates minimal rigid rotation. Rather, the video reveals an elastic wave propagating around the circumference of the sphere as the source of the nested cavities. A matryoshka cavity forms when the collision time t_c_ is longer than the elastic wave propagation time t_w_.

Supplementary Movie 3 Elasticity allows spheres to walk on water. The lift-enhancing dynamics of elastic spheres enable multiple skips over long trajectories. Modeling and laboratory observations suggest that the impact angle β_o_ decreases with each skip, which prolongs skipping despite U_o_ diminishing. This video qualitatively demonstrates this behaviour as an elastic sphere skips across a lake.
