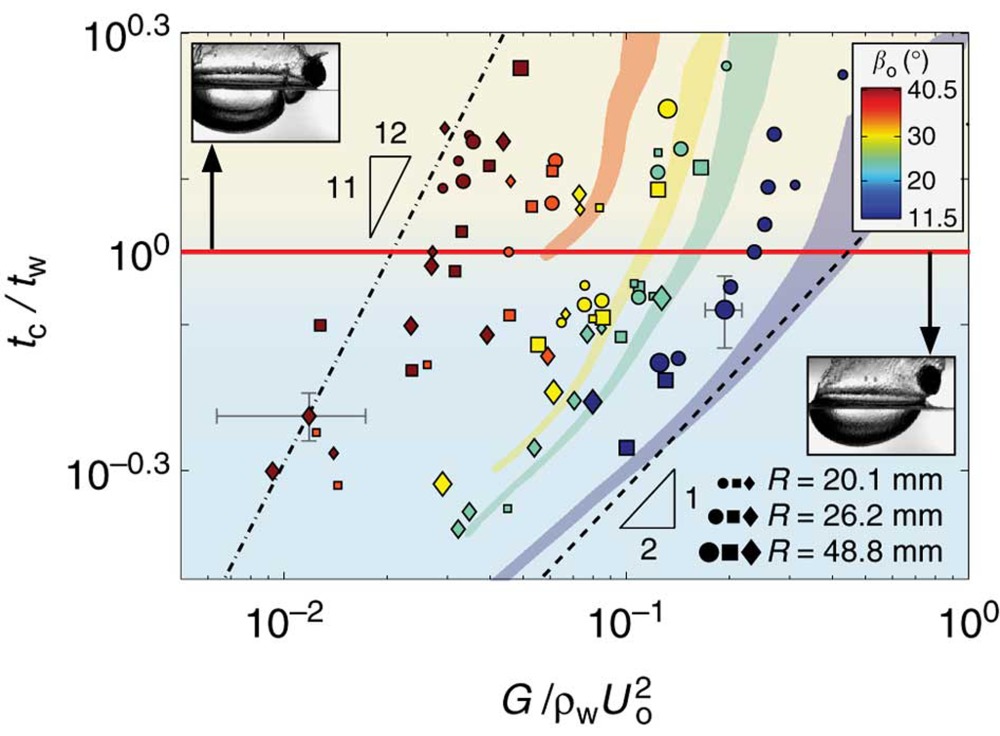# Erratum: Elastic spheres can walk on water

**DOI:** 10.1038/ncomms11679

**Published:** 2016-06-10

**Authors:** Jesse Belden, Randy C. Hurd, Michael A. Jandron, Allan F. Bower, Tadd T. Truscott

Nature Communications 7 Article number:1055110.1038/ncomms10551 (2016); Published 02042016; Updated 06102016

This Article contains an error in Fig. 5 that was introduced during the production process. The coloured background is scaled incorrectly relative to the axes and foreground figure elements. The correct version of Fig. 5 appears below.

Figure 5: