# Supplementary material for: Elastic spheres can walk on water
Source: Nat Commun. 2016 Feb 4;7:10551. doi: 10.1038/ncomms10551 (PMC4743002; doi:10.1038/ncomms10551)
Supplement: Supplementary Information — Supplementary Figures 1-3, Supplementary Notes 1-3 and Supplementary References [file ncomms10551-s1.pdf]

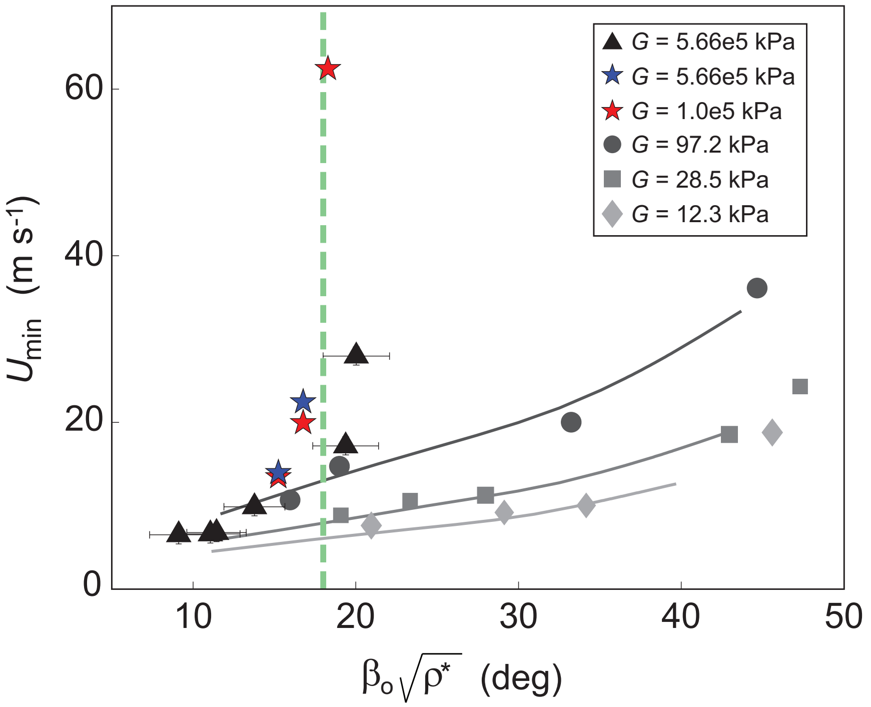

Supplementary Figure 1. Elastic spheres skip at much larger impact angles than rigid spheres. Markers result from experimental measurement with rigid spheres (black triangles: R = 25.8 mm, $\rho^* = \rho_s/\rho_w$ = 0.959) and elastic spheres (R = 26.2 mm; diamonds: ρ^* = 0.937, squares: ρ^* = 1.03, circles: ρ^* = 1.03). The coloured star markers result from Abaqus simulations (R = 26.4 mm, ρ^* = 1.03). The solid gray curves result from our analytical model (see Methods section). The vertical line denotes the predicted maximum $\beta_{o_{max}}\sqrt{\rho^*}$ = 18°, below which rigid spheres will skip. The numerical results follow this predicted bound. The experimental data seem to follow the same trend, albeit at larger values of $\beta_o\sqrt{\rho^*}$ than predicted; the bounding value of $\beta_{o_{max}}\sqrt{\rho^*}$ = 18° is nonetheless within the experimental uncertainty. The experiments also suggest a lower bound for U_{min} as $\beta_o\sqrt{\rho^*}$ decreases. This inferred value sets the lower boundary of the rigid skipping regime on Fig. 4.

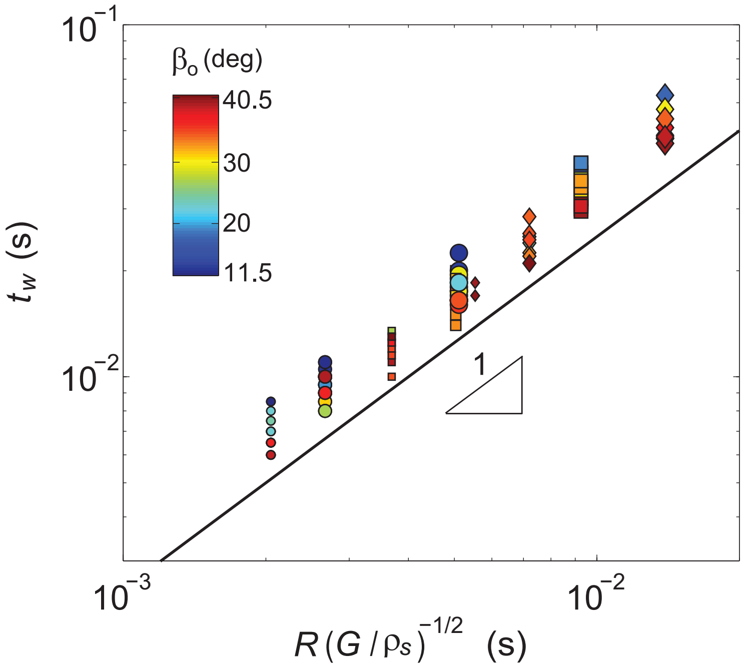

Supplementary Figure 2. The wave time is linked to the rate of change of attack angle. Our analytical model predicts that the mode 1^- elastic wave regulates $\dot{\alpha}$, which leads to the expectation that $t_\mathrm{w} \propto R/\sqrt{G/\rho_\mathrm{s}}$. This is confirmed experimentally over a range of R, G and β_o (marker shapes and sizes same as for Fig. 5). The data plotted encompass all experimental impacts characterized by the propagation of a mode 1^- type wave, including mode 1^- skipping, transitional and water entry events. Therefore, the data shown capture a range of U_o and the relatively small spread for a given set of sphere properties indicates that impact speed has a small effect on t_w.

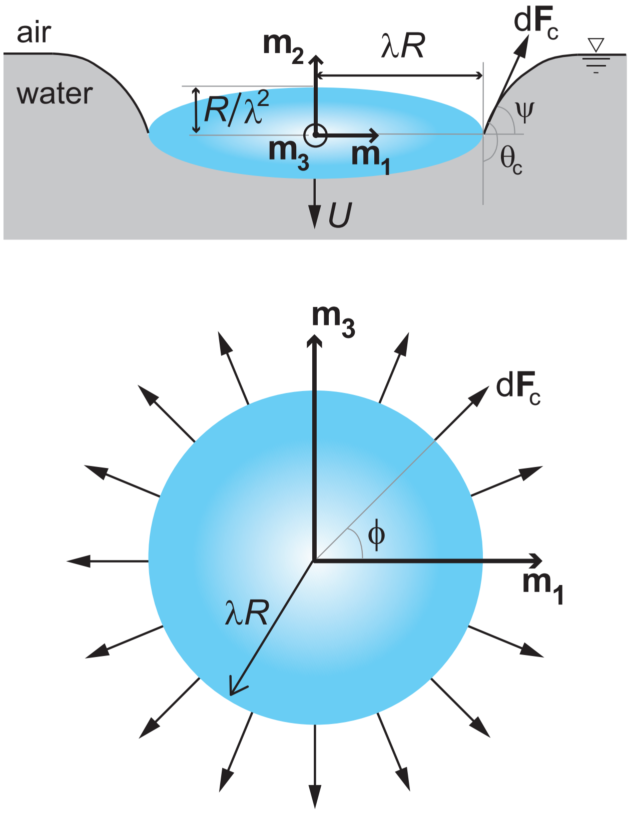

Supplementary Figure 3. Limiting case when surface tension becomes important. In the limit of $G \to 0$, and for impacts where the Weber number is of order one ($We = \rho_\mathrm{w} U_\mathrm{o}^2 R/\sigma_\mathrm{w} \approx 1$), we expect surface tension at the air-water interface to affect the sphere deformation. This simplified model shows an elastic sphere impacting normal to the air-water interface. The differential force from surface tension is dF_c and $\psi = \theta_\mathrm{c} - \pi/2$, where θ_c is the cavity cone angle. A first order force balance using our analytical model for sphere deformation leads to the expectation that surface tension effects become relevant when $G \approx \sigma_\mathrm{w}/R$ (see Supplementary Note 3).

Supplementary Notes

Supplementary Note 1: Analytical model of elastic sphere skipping

This section derives an approximate analytical approach to modeling the impact between a compliant elastomeric sphere and a fluid surface. The goal is to describe the deformation and motion using a set of reduced, scalar generalized coordinates (to be outlined) which are governed by a system of ordinary differential equations (ODEs). We begin by defining a fixed Cartesian coordinate system with $\mathbf{e_1}$ parallel to the horizontal component of velocity of the sphere, $\mathbf{e_2}$ perpendicular to the surface of the fluid, and $\mathbf{e_3}$ transverse to the motion (see Fig. 3a).

We assume that the sphere is a homogeneous, isotropic, incompressible neo-Hookean solid with radius R, shear modulus G and mass density ρ_{s}. The sphere moves parallel to the $\mathbf{e_1}$ direction and we assume it undergoes no rigid body rotation. To justify this assumption, we experimentally measured the rotational and translational kinetic energy of the sphere after exiting the water for mode 1^- skip types; we found that the rotational kinetic energy was typically less than 8% of the translational kinetic energy. The deformation of the sphere is then described as a sequence of two deformations:

1. A rigid displacement through a vector $\mathbf{d}$ that describes the motion of the center of mass (COM). The initial conditions, momentum conservation and symmetry require that $d_3 = 0$ throughout the motion.

2. A volume preserving stretch $\mathbf{V}$ that deforms the sphere into an ellipsoid. By symmetry, one of the principal directions of stretch must be parallel to the $\mathbf{e_3}$ direction. The other two principal stretch directions are parallel to unit vectors $\mathbf{m_1}, \mathbf{m_2}$, which lie in the vertical plane (see Fig. 3a). We let α be the angle between the $\mathbf{m_1}$ and $\mathbf{e_1}$ directions (positive α corresponds to rotation of the principal stretch directions about the $\mathbf{m_3}$ axis); thus α describes the attack angle of the ellipsoid. We introduce the principal stretches λ_1, λ_2 and $\lambda_3 = 1/\lambda_1\lambda_2$. The coordinates of a material particle in the sphere before deformation are given by $\mathbf{x}$, and $\mathbf{y}$ defines the coordinates after deformation.

The following identities are useful for further calculations. Elementary trigonometry shows that the unit vectors $\mathbf{e}_i$ and $\mathbf{m}_i$ are related by

$$\mathbf{e}_i = \mathbf{P}\mathbf{m}_i \tag{1}$$

where $\mathbf{P}$ is a proper orthogonal tensor whose components in both the basis $\{\mathbf{e_1}, \mathbf{e_2}, \mathbf{e_3}\}$ and $\{\mathbf{m_1}, \mathbf{m_2}, \mathbf{m_3}\}$ can be expressed as a matrix

$$[\mathbf{P}] = \begin{bmatrix} \cos\alpha & -\sin\alpha & 0 \\ \sin\alpha & \cos\alpha & 0 \\ 0 & 0 & 1 \end{bmatrix} \tag{2}$$

Since $\mathbf{e}_i$ are independent of time, derivatives of the vectors $\mathbf{m}_i$ can be calculated as

$$\dot{\mathbf{m}}_i = \frac{d}{dt}\mathbf{P}^T\mathbf{e}_i \tag{3}$$

Since $\mathbf{P}$ is orthogonal it follows that

$$\mathbf{P}\mathbf{P}^T = \mathbf{I} \implies \frac{d\mathbf{P}}{dt}\mathbf{P}^T + \mathbf{P}\frac{d\mathbf{P}^T}{dt} = 0 \implies \frac{d\mathbf{P}^T}{dt} = \mathbf{P}^T\frac{d\mathbf{P}}{dt}\mathbf{P}^T \tag{4}$$

Substituting this result into equation 3 and making use of equation 1 yields

$$\dot{\mathbf{m}}_i = \mathbf{P}^T \frac{d\mathbf{P}}{dt} \mathbf{m}_i \tag{5}$$

Evaluating this expression shows that

$$\begin{aligned} \dot{\mathbf{m}}_\mathbf{1} &= \dot{\alpha} \mathbf{m}_\mathbf{2} \\ \dot{\mathbf{m}}_\mathbf{2} &= -\dot{\alpha} \mathbf{m}_\mathbf{1} \\ \dot{\mathbf{m}}_\mathbf{3} &= 0 \end{aligned} \tag{6}$$

The standard definition of principal stretches implies that the volume preserving stretch $\mathbf{V}$ can be expressed as

$$\mathbf{V} = \lambda_1 \mathbf{m}_\mathbf{1} \otimes \mathbf{m}_\mathbf{1} + \lambda_2 \mathbf{m}_\mathbf{2} \otimes \mathbf{m}_\mathbf{2} + \frac{1}{\lambda_1 \lambda_2} \mathbf{m}_\mathbf{3} \otimes \mathbf{m}_\mathbf{3} \tag{7}$$

where $\mathbf{a} \otimes \mathbf{b}$ denotes the tensor product of two vectors; i.e the operator with the property that $[\mathbf{a} \otimes \mathbf{b}] \cdot \mathbf{c} = (\mathbf{b} \cdot \mathbf{c})\, \mathbf{a}$ for all vectors $\mathbf{c}$. Taking the time derivative of this expression and using equation 6 then shows that

$$\begin{aligned} \dot{\mathbf{V}} &= \dot{\lambda_1} \mathbf{m}_\mathbf{1} \otimes \mathbf{m}_\mathbf{1} - \frac{\dot{\lambda_1} \lambda_2 + \lambda_1 \dot{\lambda_2}}{(\lambda_1 \lambda_2)^2} \mathbf{m}_\mathbf{3} \otimes \mathbf{m}_\mathbf{3} + \dot{\lambda_2} \mathbf{m}_\mathbf{2} \otimes \mathbf{m}_\mathbf{2} \\ &+ (\lambda_1 - \lambda_2)\, \dot{\alpha} \left(\mathbf{m}_\mathbf{2} \otimes \mathbf{m}_\mathbf{1} + \mathbf{m}_\mathbf{1} \otimes \mathbf{m}_\mathbf{2} \right) \end{aligned} \tag{8}$$

and

$$\begin{aligned} \ddot{\mathbf{V}} &= \ddot{\lambda_1} \mathbf{m}_\mathbf{1} \otimes \mathbf{m}_\mathbf{1} - \frac{\ddot{\lambda_1} \lambda_2 + \lambda_1 \ddot{\lambda_2}}{(\lambda_1 \lambda_2)^2} \mathbf{m}_\mathbf{3} \otimes \mathbf{m}_\mathbf{3} + \ddot{\lambda_2} \mathbf{m}_\mathbf{2} \otimes \mathbf{m}_\mathbf{2} + (\lambda_1 - \lambda_2)\, \ddot{\alpha} \left(\mathbf{m}_\mathbf{2} \otimes \mathbf{m}_\mathbf{1} + \mathbf{m}_\mathbf{1} \otimes \mathbf{m}_\mathbf{2} \right) \\ &+ \left(\frac{2 \left(\dot{\lambda_1} \lambda_2 + \lambda_1 \dot{\lambda_2} \right)^2}{(\lambda_1 \lambda_2)^3} - \frac{2 \dot{\lambda_1} \dot{\lambda_2}}{(\lambda_1 \lambda_2)^2} \right) \mathbf{m}_\mathbf{3} \otimes \mathbf{m}_\mathbf{3} - 2 (\lambda_1 - \lambda_2)\, \dot{\alpha}^2 \left(\mathbf{m}_\mathbf{1} \otimes \mathbf{m}_\mathbf{1} - \mathbf{m}_\mathbf{2} \otimes \mathbf{m}_\mathbf{2} \right) \\ &+ \left(\dot{\lambda_1} - \dot{\lambda_2} \right) \dot{\alpha} \left(\mathbf{m}_\mathbf{2} \otimes \mathbf{m}_\mathbf{1} + \mathbf{m}_\mathbf{1} \otimes \mathbf{m}_\mathbf{2} \right) \end{aligned} \tag{9}$$

The coordinates d_1 and d_2 describe the rigid body motion and λ_1, λ_2 and α describe the deformation of the sphere. Our goal is to calculate equations of motion for these generalized coordinates. With this description we can write the deformation mapping as

$$\mathbf{y} = \mathbf{d} + \mathbf{V}\mathbf{x} \tag{10}$$

The velocity and acceleration fields follow as

$$\mathbf{v} = \dot{\mathbf{d}} + \dot{\mathbf{V}}\mathbf{x} \tag{11}$$

$$\mathbf{a} = \ddot{\mathbf{d}} + \ddot{\mathbf{V}}\mathbf{x} \tag{12}$$

We introduce a virtual velocity field

$$\delta\mathbf{v} = \delta\dot{\mathbf{d}} + \delta\dot{\mathbf{V}}\mathbf{x} \tag{13}$$

where the kinematic variables are associated with virtual rates of change $\delta\dot{d}_1$, $\delta\dot{d}_2$, $\delta\dot{\lambda}_1$, $\delta\dot{\lambda}_2$, $\delta\dot{\alpha}$ about the current (deformed) state. The governing equations for d_1, d_2, λ_1, λ_2 and α are obtained from the principle of virtual work (i.e., weak form of the momentum conservation equation)[1]

$$\int\limits_V (\sigma : \delta\mathbf{D}) dV + \int\limits_V \rho_{\mathrm{s}}(\mathbf{a}\cdot\delta\mathbf{v})dV + \int\limits_V \rho_{\mathrm{s}}(\mathbf{b}\cdot\delta\mathbf{v})dV - \int\limits_A (\mathbf{t}\cdot\delta\mathbf{v})dA = 0 \tag{14}$$

or, in index notation,

$$\int\limits_V \sigma_{ij}\delta D_{ij} dV + \int\limits_V \rho_{\mathrm{s}} a_i \delta v_i dV + \int\limits_V \rho_{\mathrm{s}} b_i \delta v_i dV - \int\limits_A t_i \delta v_i dA = 0 \tag{15}$$

where b_i represents body forces, t_i are traction forces (i.e., applied to the sphere boundary) and V and A denote integration over the volume and surface of the deformed solid, respectively. The

stretch rate in the solid is given by

$$D_{ij} = \frac{1}{2}\left(\frac{\partial v_i}{\partial y_j} + \frac{\partial v_j}{\partial y_i}\right) \tag{16}$$

The first term in equation 15 is the virtual rate of change of strain energy in the sphere, which can be calculated directly as

$$\int\limits_V \sigma_{ij}\delta D_{ij} dV = \frac{4\pi}{3} G R^3 \left(\lambda_1 \delta\dot{\lambda}_1 + \lambda_2 \delta\dot{\lambda}_2 - \frac{\lambda_1 \delta\dot{\lambda}_2 + \lambda_2 \delta\dot{\lambda}_1}{(\lambda_1\lambda_2)^3}\right) \tag{17}$$

To evaluate the remaining terms, the following identities are useful

$$\begin{aligned}\int\limits_{V_0} dV_0 &= \frac{4\pi}{3}R^3 \\ \int\limits_{V_0} x_i dV_0 &= 0 \\ \int\limits_{V_0} x_i x_j dV_0 &= \frac{4\pi}{15}R^5\delta_{ij}\end{aligned} \tag{18}$$

where x_i denote the coordinates of a material particle with respect to the center of the sphere and the integrals are evaluated over the undeformed sphere. Thus,

$$\int\limits_V \rho_s b_i \delta v_i dV = \int\limits_{V_0} \rho_s b_i \delta v_i dV_0 = -g\rho_s \frac{4\pi R^3}{3}\delta\dot{d}_2 \tag{19}$$

where we have made use of incompressibility to convert the integral over the volume of the deformed sphere (V) to an integral over the volume of the undeformed sphere (V_0). Also, we have noted that $b_i = -\rho_s g \delta_{i2}$, have substituted equation 13 for δv_i and have made use of the first two integrals in equation 18. Using equations 12-13 and again imposing incompressibility, the inertia term can be expressed as

$$\int\limits_V \rho_s a_i \delta v_i dV = \int\limits_{V_0} \rho_s a_i \delta v_i dV_0 = \int\limits_{V_0} \rho_s \left[\ddot{\mathbf{d}} + \ddot{\mathbf{V}}\mathbf{x}\right] \cdot \left[\delta\dot{\mathbf{d}} + \delta\dot{\mathbf{V}}\mathbf{x}\right] dV_0 \tag{20}$$

Expanding the terms on the right hand side yields

$$\begin{aligned}
\int_{V_0} \rho_{\mathrm{s}} \ddot{\mathbf{d}} \delta \dot{\mathbf{d}} dV_0 &= \frac{4\pi\rho_{\mathrm{s}} R^3}{3} \left(\ddot{d}_1 \delta \dot{d}_1 + \ddot{d}_2 \delta \dot{d}_2 \right) \\
\int_{V_0} \rho_{\mathrm{s}} \left[\ddot{\mathbf{V}} \mathbf{x} \right] \cdot \delta \dot{\mathbf{d}} dV_0 &= \int_{V_0} \rho_{\mathrm{s}} \ddot{\mathbf{d}} \cdot \left[\delta \dot{\mathbf{V}} \mathbf{x} \right] dV_0 = 0 \\
\int_{V_0} \rho_{\mathrm{s}} \left[\ddot{\mathbf{V}} \mathbf{x} \right] \cdot \left[\delta \dot{\mathbf{V}} \mathbf{x} \right] dV_0 &= \frac{8\pi}{15} \rho_{\mathrm{s}} R^5 \left[\ddot{\mathbf{V}} \mathbf{V} : \delta \dot{\mathbf{V}} \mathbf{V}^{-1} \right]
\end{aligned} \tag{21}$$

The nonzero terms can be interpreted physically as rates of change of translational and vibrational kinetic energies.

Finally, consider the term involving the external traction, which represents the pressure applied by the fluid on the elastomer surface,

$$\int_A t_i \delta v_i dA = \int_A \mathbf{t} \cdot \left[\delta \dot{\mathbf{d}} + \delta \dot{\mathbf{V}} \mathbf{x} \right] dA \tag{22}$$

It is preferable to express the integrand in terms of spatial coordinates. Note that from equation 10 we have $\mathbf{x} = \mathbf{V}^{-1} (\mathbf{y} - \mathbf{d})$ so that

$$\begin{aligned}
\int_A t_i \delta v_i dA &= \int_A \mathbf{t} \cdot \left[\delta \dot{\mathbf{d}} + \delta \dot{\mathbf{V}} \mathbf{V}^{-1} (\mathbf{y} - \mathbf{d}) \right] dA \\
&= \mathbf{F} \cdot \delta \dot{\mathbf{d}} + \left[\int_A \mathbf{t} \otimes (\mathbf{y} - \mathbf{d}) \, dA \right] : \left[\delta \dot{\mathbf{V}} \mathbf{V}^{-1} \right]
\end{aligned} \tag{23}$$

Here, $\mathbf{F}$ represents the resultant hydrodynamic force acting on the solid and the second term on the right hand side represents the virtual power associated with a force dipole tending to distort the elastomer.

Equation 15 can now be expressed in terms of the generalized coordinates. Substituting the expressions of equations 7-9 into equations 17,19-21 and 23, then setting each of $\delta\dot{d}_1$, $\delta\dot{d}_2$, $\delta\dot{\lambda}_1$, $\delta\dot{\lambda}_2$, $\delta\dot{\alpha}$ to be nonzero in turn will yield a set of coupled second order nonlinear ODEs. Working through this procedure yields the following governing equations:

$$\delta\dot{d}_1: \quad \frac{4\pi R^3}{3}\rho_\mathrm{s}\ddot{d}_1 = F_\mathrm{h} \tag{24}$$

$$\delta\dot{d}_2: \quad \frac{4\pi R^3}{3}\rho_\mathrm{s}\ddot{d}_2 = F_\mathrm{v} - \frac{4\pi R^3}{3}\rho_\mathrm{s} g \tag{25}$$

$$\begin{aligned}\delta\dot{\alpha}: \quad & \frac{4\pi}{15}\rho_\mathrm{s} R^5\left[2\ddot{\alpha}\left(\lambda_1-\lambda_2\right)^2\right] \\ & = \left(\lambda_1-\lambda_2\right)\int_A\left(t_1\frac{(y_2-d_2)}{\lambda_2}+t_2\frac{(y_1-d_1)}{\lambda_1}\right)dA\end{aligned} \tag{26}$$

$$\begin{aligned}\delta\dot{\lambda}_1: \quad & \frac{4\pi}{15}\rho_\mathrm{s} R^5\left[\ddot{\lambda}_1\left(1+\frac{\lambda_2^2}{(\lambda_1\lambda_2)^4}\right)+\ddot{\lambda}_2\frac{\lambda_1\lambda_2}{(\lambda_1\lambda_2)^4}-2\dot{\alpha}^2\left(\lambda_1-\lambda_2\right)+2\frac{\lambda_2\dot{\lambda}_1\dot{\lambda}_2}{(\lambda_1\lambda_2)^4}\right. \\ & \left. -2\frac{\lambda_2\left(\dot{\lambda}_1\lambda_2+\lambda_1\dot{\lambda}_2\right)^2}{(\lambda_1\lambda_2)^5}\right]+\frac{4\pi}{3}GR^3\left(\lambda_1-\frac{\lambda_2}{(\lambda_1\lambda_2)^3}\right) \\ & = \int_A\left(t_1\frac{(y_1-d_1)}{\lambda_1}-t_3\frac{(y_3-d_3)}{\lambda_1}\right)dA\end{aligned} \tag{27}$$

$$\begin{aligned}\delta\dot{\lambda}_2: \quad & \frac{4\pi}{15}\rho_\mathrm{s} R^5\left[\ddot{\lambda}_2\left(1+\frac{\lambda_1^2}{(\lambda_1\lambda_2)^4}\right)+\ddot{\lambda}_1\frac{\lambda_1\lambda_2}{(\lambda_1\lambda_2)^4}-2\dot{\alpha}^2\left(\lambda_2-\lambda_1\right)+2\frac{\lambda_1\dot{\lambda}_1\dot{\lambda}_2}{(\lambda_1\lambda_2)^4}\right. \\ & \left. -2\frac{\lambda_1\left(\dot{\lambda}_1\lambda_2+\lambda_1\dot{\lambda}_2\right)^2}{(\lambda_1\lambda_2)^5}\right]+\frac{4\pi}{3}GR^3\left(\lambda_2-\frac{\lambda_1}{(\lambda_1\lambda_2)^3}\right) \\ & = \int_A\left(t_2\frac{(y_2-d_2)}{\lambda_2}-t_3\frac{(y_3-d_3)}{\lambda_2}\right)dA\end{aligned} \tag{28}$$

where F_h and F_v are the horizontal and vertical force components in $\{\mathbf{e_1}, \mathbf{e_2}, \mathbf{e_3}\}$ coordinates, respectively; t_i and y_i are the components of the traction vector and position vector on the surface of the ellipsoid in $\{\mathbf{m_1}, \mathbf{m_2}, \mathbf{m_3}\}$ coordinates, respectively. Note that the translational degrees-of-freedom (DOF) decouple; they are coupled to vibration through the fluid, as we will show.

Before seeking a suitable expression for the hydrodynamic loading on the elastomer, we note that the governing equations predict vibration modes involving the propagation of a circumferential wave, which we have classified experimentally as mode 1^- and mode 1^+ (see Fig. 2). To see this, note that a steady-state solution exists with all tractions vanishing, and all time derivatives and second time derivatives vanishing except for the rotation rate of the $\{\mathbf{m_1}, \mathbf{m_2}, \mathbf{m_3}\}$ axes, $\dot{\alpha}$. All governing equations are satisfied trivially except for equations 27-28, which give

$$\frac{4\pi}{15}\rho_s R^5 \left[-2\dot{\alpha}^2 \left(\lambda_1 - \lambda_2\right)\right] + \frac{4\pi}{3} G R^3 \left(\lambda_1 - \frac{\lambda_2}{\left(\lambda_1 \lambda_2\right)^3}\right) = 0 \tag{29}$$

and

$$\frac{4\pi}{15}\rho_s R^5 \left[-2\dot{\alpha}^2 \left(\lambda_2 - \lambda_1\right)\right] + \frac{4\pi}{3} G R^3 \left(\lambda_2 - \frac{\lambda_1}{\left(\lambda_1 \lambda_2\right)^3}\right) = 0 \tag{30}$$

These are satisfied by any solution in which $\lambda_1 = 1/\lambda_2 = \lambda$ (other solutions are unphysical), in which case equations 29 & 30 both reduce to

$$\frac{4\pi}{15}\rho_s R^5 \left[-2\dot{\alpha}^2 \left(\lambda - \frac{1}{\lambda}\right)\right] + \frac{4\pi}{3} G R^3 \left(\lambda - \frac{1}{\lambda}\right) = 0 \tag{31}$$

The angular velocity of the axes follows as

$$\dot{\alpha} = \pm \frac{1}{R}\sqrt{\frac{5G}{2\rho_s}} \tag{32}$$

This provides the basis for the scaling of the attack angle α as measured from the Abaqus numerical simulations (Fig. 3b) as well as the scaling of the wave time t_w measured experimentally (Supplementary Fig. 2).

It is beyond the scope of this effort to derive an analytical expression for the dynamic pressure distribution over the wetted sphere surface. Rather, our goal is to generate a simplified analytical

model that captures the first order effects of an oblique water impact using the framework just derived. Even though the large degree of freedom Abaqus model has been shown to accurately capture the sphere and fluid response (see Fig. 2), a simplified model is sought to gain intuition into the dominant physics and to serve as a rapid simulation tool. Therefore, we make the following simplifying assumptions in order to specify $\mathbf{F}$ and the force dipole in terms of the shape of the elastomer and its motion:

1. The hydrodynamic force $\mathbf{F}$ is computed by considering the deformed sphere to be a circular disk described by an equivalent principal stretch λ_{eq} (to be defined), as shown in Fig. 3a. This assumption allows for computation of $\mathbf{F}$ based on an existing force model for rigid circular disk water impact (i.e., stone skipping)[2]. This model neglects viscous and surface tension forces, and is thus suitable when the Reynolds number Re = $U_{\text{o}}R/\nu \gg 1$ and Weber number We = $\rho_{\text{w}}U_{\text{o}}^2R/\sigma \gg 1$, where ν and σ are the kinematic viscosity and surface tension of water. Like skipping stones, our sphere impacts are in this inertia-dominated regime.
2. In this disk model, the resultant hydrodynamic force acts only in the $\mathbf{m_2}$ direction[2].
3. Without describing the pressure distribution, we cannot specify the center of pressure and thus cannot define the y_1 coordinate at which the traction vector acts in equation 26, which governs the attack angle α. To overcome this, we determine α from our Abaqus numerical model. With α prescribed, equations 24, 25, 27 & 28 can be solved without further simplification.

To implement the first assumption, we define λ_{eq} by equating the area of the equivalent circular disk with the cross-sectional area of the deformed sphere in the $\mathbf{m_1}$-$\mathbf{m_3}$ plane, $\pi(\lambda_{\text{eq}}R)^2 = \pi(\lambda_1 R)(\lambda_3 R)$, which gives $\lambda_{\text{eq}} = 1/\sqrt{\lambda_2}$ (Fig. 3a). We can then modify the expression for the resultant hydrodynamic force on a rigid circular disk[2] to give

$$\mathbf{F} = \frac{1}{2}\rho_{\text{w}}|\mathbf{U}_{\text{B}}|^2 S_{\text{w}} \sin(\alpha + \beta_{\text{B}})\mathbf{m_2} \tag{33}$$

where $\mathbf{u}_{\text{B}} = (u_{\text{B}}, v_{\text{B}}, w_{\text{B}})^T$ is the velocity of the point B with coordinates $(m_{1_{\text{B}}}, m_{2_B}, m_{3_B}) = (0, -\lambda_2 R, 0)$ on the bottom surface of the ball and the angle $\beta_{\text{B}} = -\tan^{-1}(v_{\text{B}}/u_{\text{B}})$ defines the instantaneous direction of $\mathbf{u}_{\text{B}}$ (Fig. 3a). The wetted area S_{w} is defined by the submerged portion of the equivalent circular disk and is computed with respect to the still free-surface. Note that α is defined as positive counter-clockwise from the $\mathbf{e_1}$ axis and β_{B} is positive clockwise from the $\mathbf{e_1}$ axis. The force is assumed to act through the COM of the deformed sphere and is non-zero only when $\beta_{\text{B}} > -\alpha$.

Next, we express $|\mathbf{U}_{\text{B}}|$, β_{B} and S_{w} in terms of the kinematics of the deformed ball. Evaluating equation 11 gives the velocity of point B in $\{\mathbf{e_1}, \mathbf{e_2}, \mathbf{e_3}\}$ coordinates,

$$\mathbf{U}_{\text{B}} = \dot{\mathbf{d}} + \mathbf{P}\dot{\mathbf{V}}\mathbf{x}_{\text{B}} \tag{34}$$

where $\mathbf{x}_{\text{B}} = (0, -R, 0)^T$ in $\{\mathbf{m_1}, \mathbf{m_2}, \mathbf{m_3}\}$ coordinates and multiplication by $\mathbf{P}$ takes $\dot{\mathbf{V}}\mathbf{x}_{\text{B}}$ to

$\{\mathbf{e_1}, \mathbf{e_2}, \mathbf{e_3}\}$ coordinates. Applying the identities in equations 1-9 gives

$$\mathbf{U}_\mathrm{B} = \begin{pmatrix} u_\mathrm{B} \\ \\ v_\mathrm{B} \\ \\ w_\mathrm{B} \end{pmatrix} = \begin{pmatrix} \dot{d}_1 - R\left(\dot{\alpha}\left(\lambda_1 - \lambda_2\right)\cos\alpha - \dot{\lambda_2}\sin\alpha\right) \\ \\ \dot{d}_2 - R\left(\dot{\alpha}\left(\lambda_1 - \lambda_2\right)\sin\alpha + \dot{\lambda_2}\cos\alpha\right) \\ \\ 0 \end{pmatrix} \tag{35}$$

The angle of the velocity vector at B is then

$$\beta_\mathrm{B} = -\tan^{-1}\left(\frac{v_\mathrm{B}}{u_\mathrm{B}}\right) = -\tan^{-1}\left(\frac{\dot{d}_2 - R\left(\dot{\alpha}\left(\lambda_1 - \lambda_2\right)\sin\alpha + \dot{\lambda_2}\cos\alpha\right)}{\dot{d}_1 - R\left(\dot{\alpha}\left(\lambda_1 - \lambda_2\right)\cos\alpha - \dot{\lambda_2}\sin\alpha\right)}\right) \tag{36}$$

The wetted area follows from the derivation for a rigid disk[2],

$$S_\mathrm{w} = \left(\lambda_\mathrm{eq} R\right)^2 \left[\cos^{-1}\left(1 - \frac{s}{\lambda_\mathrm{eq} R}\right) - \left(1 - \frac{s}{\lambda_\mathrm{eq} R}\right)\sqrt{1 - \left(1 - \frac{s}{\lambda_\mathrm{eq} R}\right)^2}\,\right] \tag{37}$$

where s is the wetted length in the $\mathbf{e_1}$-$\mathbf{e_2}$ plane (Fig. 3a) defined as

$$s = \begin{cases} 0 & \text{if } d_2 \geq R\left(\lambda_\mathrm{eq}\sin\alpha + \lambda_2\cos\alpha\right) \\ \\ -d_2/\sin\alpha + R\left(\lambda_\mathrm{eq} + \lambda_2\cot\alpha\right) & \text{else} \\ \\ 2\lambda_\mathrm{eq} R & \text{if } d_2 \leq -R\left(\lambda_\mathrm{eq}\sin\alpha - \lambda_2\cos\alpha\right) \end{cases} \tag{38}$$

Finally, to complete the simplified model we prescribe α based on the results from simulations using our numerical Abaqus model over a range of U_o and β_o with the following sphere properties: $R = 25.4$ mm, ρ^* = 1.05 and varying shear modulus ($G = 12.3$, 28.5 and 97.2 kPa). Recognizing that this approach is only useful if a general model for α can be established, we seek a suitable scaling of the data from simulations of mode 1^- skipping and mode 1^- transitional events. Given the angular rate of mode 1^- predicted by equation 32, we expect the attack angle to scale as

$\alpha \propto t\frac{1}{R}\sqrt{\frac{G}{\rho_s}} \equiv t^*$. Figure 3b shows good collapse using this scaling, particularly for $\beta_o \leq 29.5°$. For these angles, we apply the following piecewise linear model for α:

$$\alpha = \begin{cases} \frac{A_1 \sin\beta_o}{t_1^*} t^* & t^* < t_1^* \\ A_2\left(t^* - t_1^*\right) + A_1 \sin\beta_o & t_1^* \leq t^* < t_2^* \\ A_3\left(t^* - t_2^*\right) + A_2\left(t_2^* - t_1^*\right) + A_1 \sin\beta_o & t_2^* \leq t^* < t_3^* \\ A_4 \sin\beta_o\left(t^* - t_3^*\right) + A_3\left(t_3^* - t_2^*\right) + A_2\left(t_2^* - t_1^*\right) + A_1 \sin\beta_o & t^* > t_3^* \end{cases} \tag{39}$$

where the coefficients A_i are found from fits to the data (A_1 = 0.332, A_2 = 0.446, A_3 = 0.118, A_4 = 0.686) in each region defined by the dimensionless times t_1^* = 0.15, t_2^* = 1 and t_3^* = 2. The first and last regions are β_o-dependent, while the middle two regions are independent of β_o but feature a slope change at t^* = 1. Applying this fit to the data shown in Fig. 3b, we find a standard error of the fit of $\pm$2.35°.

For simulation data with $\beta_o \geq 34.5°$, we find a dependence on $G^* = G/\rho_w U_o^2$ for $t_1^* \leq t^* < t_2^*$. For these angles, the piecewise linear model for α is given by,

$$\alpha = \begin{cases} \frac{A_1 \sin\beta_o}{t_1^*} t^* & t^* < t_1^* \\ \left(A_2 + C_2 \sin\beta_o / G^{*^a}\right)\left(t^* - t_1^*\right) + A_1 \sin\beta_o & t_1^* \leq t^* < t_2^* \\ C_3\left(t^* - t_2^*\right) + \left(A_2 + C_2 \sin\beta_o / G^{*^a}\right)\left(t_2^* - t_1^*\right) + A_1 \sin\beta_o & t_2^* \leq t^* < t_3^* \\ C_4\left(t^* - t_3^*\right) + C_3\left(t_3^* - t_2^*\right) + \left(A_2 + C_2 \sin\beta_o / G^{*^a}\right)\left(t_2^* - t_1^*\right) + A_1 \sin\beta_o & t^* > t_3^* \end{cases} \tag{40}$$

where the parameters are found from fits to the data in each region defined by the same dimensionless times as used for equation 39 (a = 1.04, C_2 = 0.0219, C_3 = 0.108, C_4 = 0.324). The standard

error of this model fit to the numerical data is $\pm 4.81°$.

The value of α and $\dot{\alpha}$ in equations 27, 28, 33 & 35-38 are computed using the models in equations 39 & 40. As our numerical simulations leave a gap for $29.5° < \beta_{\mathrm{o}} < 34.5°$, we use equation 39 for $\beta_{\mathrm{o}} \leq 32°$ and equation 40 else.

We now insert the hydrodynamic force (equation 33) into the general equations of motion (equations 24,25,27 & 28). Inserting the force components $F_{\mathrm{h}} = -|\mathbf{F}| \sin\alpha$ and $F_{\mathrm{v}} = |\mathbf{F}| \cos\alpha$ into equations 24 & 25 gives

$$\ddot{d}_1 = -\frac{3}{8\pi R^3} \frac{\rho_{\mathrm{w}}}{\rho_{\mathrm{s}}} |\mathbf{U}_{\mathrm{B}}|^2 S_{\mathrm{w}} \sin(\alpha + \beta_{\mathrm{B}}) \sin\alpha \tag{41}$$

$$\ddot{d}_2 = \frac{3}{8\pi R^3} \frac{\rho_{\mathrm{w}}}{\rho_{\mathrm{s}}} |\mathbf{U}_{\mathrm{B}}|^2 S_{\mathrm{w}} \sin(\alpha + \beta_{\mathrm{B}}) \cos\alpha - g \tag{42}$$

Per our assumption that the only traction is in the $\mathbf{m_2}$ direction, equation 27 reduces to

$$\frac{4\pi}{15}\rho_{\mathrm{s}} R^5 \left[\ddot{\lambda}_1 \left(1 + \frac{\lambda_2^2}{(\lambda_1\lambda_2)^4} \right) + \ddot{\lambda}_2 \frac{\lambda_1\lambda_2}{(\lambda_1\lambda_2)^4} - 2\dot{\alpha}^2 (\lambda_1 - \lambda_2) + 2\frac{\lambda_2 \dot{\lambda}_1 \dot{\lambda}_2}{(\lambda_1\lambda_2)^4} - 2\frac{\lambda_2 \left(\dot{\lambda}_1 \lambda_2 + \lambda_1 \dot{\lambda}_2 \right)^2}{(\lambda_1\lambda_2)^5} \right] + \frac{4\pi}{3} G R^3 \left(\lambda_1 - \frac{\lambda_2}{(\lambda_1\lambda_2)^3} \right) = 0 \tag{43}$$

To express the forcing term in equation 28, we apply the point force $\mathbf{F} = |\mathbf{F}|\mathbf{m_2}$ at $y_2^* = d_2 - \lambda_2 R$. The right-hand side of equation 28 can be written as

$$\int_A t_2 \frac{y_2 - d_2}{\lambda_2} dA = \int_A |\mathbf{F}| \delta\left(y_2 - y_2^*\right) \frac{y_2 - d_2}{\lambda_2} dA = |\mathbf{F}| \frac{y_2^* - d_2}{\lambda_2} = -|\mathbf{F}| R \tag{44}$$

Combining this result with equations 28 & 33 gives

$$\frac{4\pi}{15}\rho_{\mathrm{s}}R^4\left[\ddot{\lambda}_2\left(1+\frac{\lambda_1^2}{(\lambda_1\lambda_2)^4}\right)+\ddot{\lambda}_1\frac{\lambda_1\lambda_2}{(\lambda_1\lambda_2)^4}-2\dot{\alpha}^2\left(\lambda_2-\lambda_1\right)+2\frac{\lambda_1\dot{\lambda}_1\dot{\lambda}_2}{(\lambda_1\lambda_2)^4}-2\frac{\lambda_1\left(\dot{\lambda}_1\lambda_2+\lambda_1\dot{\lambda}_2\right)^2}{(\lambda_1\lambda_2)^5}\right]$$
$$+\frac{4\pi}{3}GR^3\left(\lambda_2-\frac{\lambda_1}{(\lambda_1\lambda_2)^3}\right)=-\frac{1}{2}\rho_{\mathrm{w}}|\mathbf{U}_{\mathrm{B}}|^2S_{\mathrm{w}}\sin(\alpha+\beta_{\mathrm{B}}) \quad (45)$$

The four governing ODEs (equations 41-43 & 45) are solved using a fourth-order Runge-Kutta solver in Matlab, with $\mathbf{U}_{\mathrm{B}}$, β_{B} and S_{w} defined by equations 35-38, and α, $\dot{\alpha}$ defined by either equation 39 or 40, depending on the value of β_{o}.

Supplementary Note 2: Typical values and scaling analysis

As shown in the Methods section, a scaled version of equation 42 can be estimated as

$$\frac{2R}{t_{\mathrm{c}}^2}\approx\frac{3}{8\pi R\rho^*}U_{\min}^2\lambda_{\max}^2\sin(\alpha+\beta_{\mathrm{B}})\cos\alpha-g \quad (46)$$

We examine terms in equation 46 using representative values for a sphere that barely skips and has small G for which we expect $\dot{\alpha}\propto\frac{1}{R}\sqrt{\frac{G}{\rho_{\mathrm{s}}}}\rightarrow 0$. A typical simulation using the analytical model with a relatively small shear modulus $G=1$ kPa and sphere properties $R=26.4$ mm and $\rho_{\mathrm{s}}=1032$ kg m^{-3} gives a mean value of $\cos\alpha=0.68$ over the duration of impact. Furthermore, the mean value of $\sin(\alpha+\beta_{\mathrm{B}})=0.64$ and thus both angle dependent terms are of order 1. For threshold skipping cases, we expect the characteristic acceleration $2R/t_{\mathrm{c}}^2$ to be small compared to gravity (see Methods) and thus predict $3U_{\min}^2\lambda_{\max}^2/8\pi R\rho^*\approx g$, which gives $U_{\min}\approx\sqrt{8\pi g\rho^*R/3\lambda_{\max}^2}$. Using the typical values from the simulation case, for which $\lambda_{\max}=1.44$, gives $U_{\min}=1.04$ m s^{-1}, which is on the same order as the actual impact velocity used to generate the simulation, $U_o=$

2.71 m s^{-1}.

We now consider typical values of the terms in equation 46 for skipping events in which $t_c \approx t_w$. We consider a representative experiment with R = 26.2 mm, G = 12.3 kPa, ρ^* = 0.937, U_o = 13.2 m s^{-1} and β_o = 24.2° that results in skipping with measured t_c = 15 ms and t_w = 24 ms. Simulation with our analytical model using these same parameters reveals a mean value of $\sin(\alpha + \beta_B)\cos\alpha \approx$ 0.4 and $\lambda_{max} \approx$ 1.5. Thus $1/\sqrt{\sin(\alpha + \beta_B)\cos\alpha} \approx 1$ and based on the scaling analysis (see Methods section), we expect $t_c \approx \frac{R}{U_o\lambda_{max}}\sqrt{\frac{16\pi\rho^*}{3}}$. Inserting the experimental values into this expression gives $t_c \approx$ 8 ms, which is comparable to the actual measured collision time of t_c = 15 ms. Thus, the scaling for the collision time is $t_c \propto (R/U_o\lambda_{max})\sqrt{\rho^*}$. Combining this with the wave time scaling, $t_w \propto R/\sqrt{G/\rho_s}$ (see Supplementary Fig. 2), gives the expected dependence of the timescale ratio,

$$\frac{t_c}{t_w} \propto \left(\frac{G}{\rho_w U_o^2}\right)^{1/2} \frac{1}{\lambda_{max}} \tag{47}$$

In the shallow β_o limit, $t_c/t_w \approx 1$ occurs at values of $G/\rho_w U_o^2 > 0.1$. For example, for $\beta_o = 11.5°$, $t_c/t_w = 1$ occurs at $G/\rho_w U_o^2 = 0.24$ (see Fig. 5) and for shallower impact angles we expect this critical value of $G/\rho_w U_o^2$ to continue to increase. As $G/\rho_w U_o^2$ increases, we expect $\lambda_{max} \to 1$ (Fig. 3c) and thus anticipate $t_c/t_w \propto (G/\rho_w U_o^2)^{1/2}$ for shallow angles. For steeper values of β_o (e.g., $\beta_o > 34.5°$), our simulations indicate a limiting dependence of $\lambda_{max} \propto (G/\rho_w U_o^2)^{-5/12}$ for critical values of $G/\rho_w U_o^2$ for which $t_c/t_w = 1$ (Fig. 3c, Fig. 5). Therefore, in the steep β_o limit we expect $t_c/t_w \propto (G/\rho_w U_o^2)^{11/12}$.

Supplementary Note 3: Small stiffness limit in which surface tension is important.

We now estimate the value of shear modulus at which surface tension forces have a non-negligible effect on deformation by considering an elastic sphere impacting normal to the surface ($\beta_{\mathrm{o}} = 90°$, see Supplementary Fig. 3). First, we expect surface tension to be important to the overall dynamics of sphere impact (rigid or elastic) when the Weber number is of order one, $We = \rho_{\mathrm{w}} U_{\mathrm{o}}^2 R/\sigma_{\mathrm{w}} \approx 1$. We assume symmetry in the sphere deformation such that $\lambda_1 = \lambda_3 = \lambda$ and $\lambda_2 = 1/\lambda^2$. The components of the differential force from surface tension are $dF_{c_1} = \sigma_{\mathrm{w}} \cos\psi(\lambda R d\phi)\cos\phi$, $dF_{c_2} = \sigma_{\mathrm{w}} \sin\psi(\lambda R d\phi)$ and $dF_{c_3} = \sigma_{\mathrm{w}} \cos\psi(\lambda R d\phi)\sin\phi$, where $\psi = \theta_{\mathrm{c}} - \pi/2$ and θ_{c} is the cavity cone angle[3]. In the limit of $G \to 0$, we expect the period of sphere vibrations $t_{\mathrm{w}} \to \infty$ such that $\ddot{\lambda}$ and $\dot{\lambda}$ are negligible. Considering equation 28 with all time derivatives set to zero gives

$$\frac{4\pi}{3} G R^3 \left(\frac{1}{\lambda^2} - \lambda^4\right) = \int_A \left(t_2 \lambda^2 \left(y_2 - d_2\right) - t_3 \lambda^2 \left(y_3 - d_3\right)\right) dA \tag{48}$$

For a first order analysis, we assume the surface tension force acts at $y_2 - d_2 = 0$, which gives

$$\frac{4\pi}{3} G R^3 \left(\lambda^4 - \frac{1}{\lambda^2}\right) = \sigma_{\mathrm{w}} \cos\theta \lambda^3 R^2 \int_0^{2\pi} \sin^2\phi d\phi \tag{49}$$

Solving equation 49 and rearranging gives

$$\lambda - \frac{1}{\lambda^5} = \frac{3\sigma_{\mathrm{w}} \cos\theta}{4GR} \tag{50}$$

For the left-hand side of equation 50 to be non-zero requires $\sigma_{\mathrm{w}}/GR \approx 1$, which gives $G \approx \sigma_{\mathrm{w}}/R$.

Supplementary References

1. Bower, A. F. *Applied mechanics of solids* (CRC press, 2009).

2. Rosellini, L., Hersen, F., Clanet, C. & Bocquet, L. Skipping stones. *J. Fluid Mech.* **543**, 137–146 (2005).

3. Aristoff, J. & Bush, J. Water entry of small hydrophobic spheres. *J. Fluid Mech.* **619**, 45–78 (2009).
